# Achieving Anti‐Disproportionation Performance Enhancement and Distorted Inverse‐Disproportionation Reaction Correction of Zr_2_Fe‐Based Hydrogen Isotope Storage Alloys via Element Substitution

**DOI:** 10.1002/advs.202507722

**Published:** 2025-06-29

**Authors:** Zhiyi Yang, Yuxiao Jia, Yang Liu, Fei Chu, Jiacheng Qi, Tiao Ying, Jiahuan He, Xingwen Feng, Jiangfeng Song, Yan Shi, Wenhua Luo, Xuezhang Xiao, Lixin Chen

**Affiliations:** ^1^ State Key Laboratory of Silicon and Advanced Semiconductor Materials School of Materials Science and Engineering Zhejiang University Hangzhou 310058 China; ^2^ Key Laboratory of Hydrogen Storage and Transportation Technology of Zhejiang Province Hangzhou 310027 China; ^3^ Institute of Materials China Academy of Engineering Physics Mianyang 621907 China; ^4^ School of Advanced Energy Sun Yat‐Sen University Shenzhen Guangdong 518107 China

**Keywords:** alloying modification, anti‐disproportionation properties, inverse disproportionation reaction, tritium‐getter materials, Zr_2_Fe‐based alloys

## Abstract

Zr_2_Fe alloy is a promising candidate as a tritium‐getter material for the International Thermonuclear Experimental Reactor (ITER), but its practical application is hindered by undesirable inverse disproportionation behavior and poor anti‐disproportionation properties. In this study, theoretical computational screening is utilized to predict the effects of partially substituting Fe with Co, Cu, and Ni on regulating the Zr_2_Fe_1‐x_M_x_ (M = Co, Cu, Ni; x = 0.1–0.5) hydrogen storage systems. Experimentally, these modifications successfully correct the distorted inverse disproportionation reaction and achieve full reversibility in the Zr_2_Fe_1‐x_M_x‐_H systems. Notably, Zr_2_Fe_0.8_Cu_0.2_ and Zr_2_Fe_0.7_Ni_0.3_ alloys retain excellent hydrogen storage properties, while their kinetic energy barriers of hydriding disproportionation reaction increase significantly from 87.88 kJ mol^−1^ (Zr_2_Fe) to 184.35 kJ mol^−1^ (Zr_2_Fe_0.8_Cu_0.2_) and 192.32 kJ mol^−1^ (Zr_2_Fe_0.7_Ni_0.3_), respectively. The corresponding deceleration of hydriding disproportionation kinetics behaviors is clearly visualized by TEM observations. Combined density functional theory analyses reveal that the mechanism underlying enhanced anti‐disproportionation properties in the optimized Zr_2_Fe_1‐x_M_x‐_H systems involves the homogenization and stabilization of Zr─H bonds within the hydrogen storage interstices, along with the effective suppression of disproportionation‐favorable chemical environments.

## Graphical abstract

Undesirable phase transition behavior and anti‐disproportionation performance limit the hydrogen isotope storage of Zr_2_Fe alloy. With theoretical screening and experiments, partial Cu/Ni substitutions achieve full reversibility and significantly improve anti‐disproportionation properties. Modulation of charge redistribution and chemical environment, combined with thermodynamic suppression, synergistically enhance resistance to disproportionation.

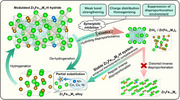

## 1 Introduction

With the growing global energy demand and the gradual depletion of fossil fuel resources, exploring advanced energy technologies has become essential. The International Thermonuclear Experimental Reactor (ITER), a leading deuterium‐tritium fusion project for clean energy production, is widely regarded as a promising solution to the energy crisis.^[^ ^1^ ^]^ Due to the frequent delivery of precious fuels, the development of tritium capture and recovery technology to ensure the stability of expanding reactors has attracted significant attention in recent years. Solid‐state hydrogen isotope storage technology is a safe and effective method for storing radioactive tritium, owing to its excellent stability and suitable operating conditions.^[^ ^2^ ^]^ Among them, the Zr_2_X (X = Ni, Co, Fe) alloys, due to their low equilibrium hydrogen pressure and fast hydrogen absorption kinetics, are capable of efficiently capturing low concentrations of tritium.^[^ ^3^ ^]^

Zr_2_Fe alloy exhibits an extremely low hydrogenation equilibrium pressure and excellent hydriding kinetics. Notably, its room‐temperature equilibrium hydrogen pressure (2.68 × 10^−8^ Pa) is over two orders of magnitude lower than that of Zr_2_Ni, Zr_2_Co, and ZrCo, allowing Zr_2_Fe to be considered as one of the most promising getter materials for treating tritium in Ci or gram scale.^[^ ^4^, ^5^ ^]^ Moreover, Zr_2_Fe possesses a distinct advantage over Zr_2_Co and Zr_2_Ni. The dehydrogenation of Zr_2_CoH_5_ always leads to the formation of ZrCoH_3_ and ZrH_2_ phases. Similarly, Zr_2_NiH_5_ decomposes preferentially into ZrNiH_3_ and ZrH_2_ during the dehydrogenation process.^[^ ^5^, ^6^ ^]^ In contrast, Zr_2_Fe does not undergo unconventional decomposition reactions that readily form disproportionation phases during dehydrogenation, which is advantageous for its durability over multiple de‐/hydrogenation cycling.

Nevertheless, the inherent tendency toward hydriding disproportionation remains a fundamental characteristic of Zr‐based alloys, particularly under high temperatures. This intrinsic behavior significantly undermines their hydrogen storage performance and reliability during cyclic applications.^[^ ^2^, ^7^ ^]^ Prigent et al.^[^ ^8^ ^]^ found that the hydrogenation capacity of Zr_2_Fe in the third cycle was only 0.84 wt.% during cycling at desorption at 350 °C, with a sharp degradation compared to the previous cycle (1.18 wt.%), reflecting the susceptibility of Zr_2_Fe to disproportionation at high temperatures. Hara et al.^[^ ^9^ ^]^ presented an overview of experimental observations for the high‐temperature disproportionation of Zr_2_X (X = Ni, Co, Fe), and also found that in the case of disproportionation, the binary Zr‐based A_2_B alloy transforms into ZrH_2_ and a new B‐rich metallic compound. The ease of disproportionation differs from one alloy to another, and the disproportionation behavior is considerably affected by the B‐side element. The hydriding disproportionation phenomenon is more pronounced in the Zr_2_X series alloys than in ZrCo alloys, yet current anti‐disproportionation studies on Zr_2_X alloys are even more immature. For instances, Komeili et al.^[^ ^10^ ^]^ prepared the Zr_2_(Co_0.5_Fe_0.2_Ni_0.2_V_0.1_) alloy to enhance certain cycling performance relative to the Zr_2_Co alloy, validating the demonstration that it is feasible to improve the disproportionation resistance of Zr_2_Co alloys via alloying. Xie et al.^[^ ^11^ ^]^ conducted a high‐throughput computational study on the effects of Ni and Ti doping on the structural stability of Zr_2_Fe, with the expectation of providing a reference for improving its hydrogen absorption and anti‐disproportionation performance. However, relevant studies relied solely on trial‐and‐error experiments or theoretical calculations, which could not reveal the modification mechanism of alloying methods on Zr_2_X alloys. The lack of understanding of performance enhancement fails to provide practical and effective guidance for the compositional design of Zr_2_X alloys to further improve their overall performance. Our previous study investigated the hydrogen storage and disproportionation mechanisms of Zr_2_Co and Zr_2_Fe alloys in a combined experimental and theoretical approach.^[^ ^5^, ^6^ ^]^ Based on these findings, we have designed the Zr_1.8_Hf_0.2_Co_0.8_Fe_0.1_Ni_0.1_ alloy to improve disproportionation resistance and discovered a propensity rule between the disorganization of hydrogen binding energy at various interstices and anti‐disproportionation performance.^[^ ^12^ ^]^ However, there is still significant room for improvement in the understanding of the deep underlying mechanisms by which alloying modifications affect the comprehensive hydrogen storage and anti‐disproportionation performance of Zr_2_X alloy systems, particularly in the Zr_2_Fe system.

Furthermore, the Zr_2_Fe alloy suffers from a critical defect in that the disproportionation products ZrH_2_ and ZrFe_2_ undergo a stabilization‐driven transformation into Zr_3_Fe and residual ZrFe_2_ during thermal dehydrogenation at 700 °C (distorted inverse disproportionation reaction), whereas Zr_2_X alloys can be reconstructed to original state in the Zr_2_Co‐H and Zr_2_Ni‐H systems (regular inverse disproportionation reaction). The Zr_3_Fe alloy exhibits a hydrogen absorption ability similar to that of Zr_2_Fe, with a practical hydrogen storage capacity of 1.88 wt.% at room temperature. However, hydrogen desorption is considerably restricted in the Zr_3_Fe‐H system, and less than 0.72 wt.% (38% of saturation capacity) can be released after treatment at 390 °C.^[^ ^13^ ^]^ Furthermore, Zr_3_Fe is found to be more susceptible to disproportionation, as evidenced by the formation of a significant amount of ZrH_2_ under a hydrogen environment as low as 200 °C.^[^ ^14^ ^]^ Therefore, the presence of Zr_3_Fe should be strictly avoided in Zr_2_Fe‐based systems due to its extremely poor cycling performance. In addition, the irreversibility of the Zr_2_Fe‐H system severely limits certain microstructural modification strategies and imposes constraints on its full activation and refurbishment, as mentioned in many works.^[^ ^15^, ^16^, ^17^ ^]^

The differences in the B‐side elements have a dominant impact on the phase transition for Zr_2_X‐H systems. With regard to the above drawbacks of Zr_2_Fe, this work conducts a targeted screening of alloying elements in terms of energy variations, thereby effectively correcting the distorted inverse disproportionation reaction and achieving full reversibility. Through experimental verifications of the effectiveness with different substitutions of Fe with Co, Ni, and Cu, combined with analysis of hydriding disproportionation reaction kinetics, the Zr_2_Fe‐based alloy is shown to have significantly enhanced resistance to hydriding disproportionation, while retaining its ultra‐low equilibrium hydrogen pressure and superior hydrogenation kinetics. The beneficial patterns of Fe‐side alloying in inhibiting the hydriding disproportionation reaction are clearly revealed. Furthermore, focusing on the preferred Zr_2_Fe_1‐_ *_x_* M*_x_*‐H systems, the microscopic modulation effect of alloying in the Zr_2_Fe_1‐_ *_x_* M*_x_*‐H systems is confirmed in combination with density functional theory (DFT) calculations of crystal structure, removal energy, bonding strength, and electronic distribution, consequently revealing the modification mechanism of the enhanced anti‐disproportionation performance.

## 2 Results and Discussion

**Figure 1:**
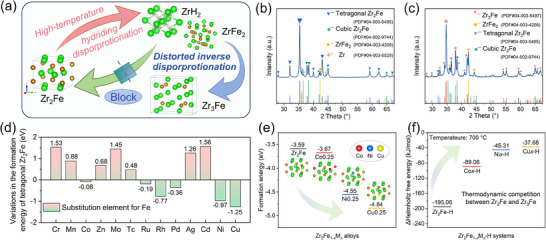
The schematic illustration of disproportionation and inverse disproportionation in Zr_2_Fe‐H system (a); XRD patterns of Zr_2_Fe alloy (b) and inverse disproportionation product (c); Formation energy variations of tetragonal Zr_2_Fe alloy with different substitution elements for Fe site (d); Formation energy of Zr_2_Fe and Zr_2_Fe_0.75_M_0.25_ alloys (e); Helmholtz free energy differences (Δ*F'*) between the products of two inverse disproportionation pathways at 700 °C in Zr_2_Fe_1‐_ *_x_* M*_x_*‐H systems (f).

Regarding the issue of inverse disproportionation, as shown schematically (**Figure** **1**a), the Zr_2_Fe‐H system can undergo a hydriding disproportionation reaction driven by high temperatures, resulting in the formation of ZrH_2_ and ZrFe_2_. To restore the system and refurbish the hydrogen storage performance, the temperature can be increased to 700 °C for the decomposition of ZrH_2_. However, during this process, the ZrH_2_ and ZrFe_2_ cannot be reconfigured back to Zr_2_Fe, instead basically being converted to Zr_3_Fe, which can be described as a distorted inverse disproportionation reaction. The whole reaction equation is given as follows:

(1) $$Zr_{2}\mathrm{Fe}\;+H_{2}\to \;\mathrm{Zr}H_{2}+\;\mathrm{ZrF}e_{2}\to \;Zr_{3}\mathrm{Fe}\;+\;\mathrm{ZrF}e_{2}+H_{2}$$

The as‐cast Zr_2_Fe alloy and its inverse disproportionation product in the Zr_2_Fe‐H system were characterized by X‐ray diffraction (XRD). The Zr_2_Fe alloy mainly consists of tetragonal Zr_2_Fe, with a small amount of cubic Zr_2_Fe and trace amounts of segregated ZrFe_2_ and Zr (Figure 1b). As depicted in Figure 1c, the most prominent diffraction peaks at 34.74° and 37.91° correspond to the Zr_3_Fe phase (PDF#04‐003‐5497), and a distinct diffraction peak at 42.27° corresponds to the ZrFe_2_ phase (PDF#04‐003‐4208), while the characteristic diffraction peak of Zr_2_Fe at 35.32° is almost undetectable. The results suggest that the hydriding disproportionation products ZrH_2_ and ZrFe_2_ react to form Zr_3_Fe and H_2_ at 700 °C, leaving a partial amount of ZrFe_2_ unreacted. This provides convincing evidence for the preferential growth of the Zr_3_Fe crystallites and the relatively high formation energy of the thermodynamically less stable Zr_2_Fe, as also reflected by a narrow region of the Zr_2_Fe compound in the Zr‐Fe phase diagram.^[^ ^18^, ^19^ ^]^

Based on this, a computational screening of the transition elements in the fourth and fifth periods was performed to explore strategies for stabilizing the Zr_2_Fe phase (Figure 1d). The formation energy variation during partial substitution on the Fe side was determined as the evaluation index to assess the formation ability of the body‐centered tetragonal Zr_2_Fe_1‐_ *_x_* M*_x_* phase.^[^ ^20^ ^]^ Among the substitution elements, Cr, Mn, Zn, Mo, Tc, Ag, and Cd all increase the formation energy of the tetragonal Zr_2_Fe phase, leading to thermodynamic instability. Of these, Cr and Mn, which are commonly used in hydrogen storage alloys, were selected to investigate their substitution effects.^[^ ^21^, ^22^ ^]^ As shown in Figure S1 (Supporting Information), both Zr_2_Fe_0.8_Cr_0.2_ and Zr_2_Fe_0.8_Mn_0.2_ could not maintain the original tetragonal phase, showing significant changes in the phase composition. Favorable substitution elements Cu and Ni exhibit a relatively large reduction in the formation energy of Zr_2_Fe, followed by Rh, Pd, Ru, and Co. Furthermore, due to cost and hydrogen weight density considerations, Rh, Pd, and Ru are not recommended. Following the screening, Cu, Ni, and Co emerge as potential substituents to enhance the thermodynamic stability of the Zr_2_Fe phase. Figure 1e presents the formation energy values after substitution, exhibiting that 0.25 atomic substitution of Cu and Ni reduces the formation energy of the Zr_2_Fe phase from −3.59 to −4.84, and −4.55 eV, respectively, and the Co substitution leads to a reduction of only 0.08 eV.

To address the deficiency of inverse disproportionation, the concern is whether the latter stage of Reaction (1) can be altered to a regular inverse disproportionation reaction:

(2) $$\mathrm{Zr}H_{2}+\;\mathrm{ZrF}e_{2}\to \;Zr_{2}\mathrm{Fe}\;+H_{2}$$

Furthermore, the calculations of the Helmholtz free energy (Δ*F*) at constant temperature and constant volume were introduced to evaluate the competitive relation between Reaction (1) and Reaction (2) at 700 °C in Zr_2_Fe‐H, Cu*x*‐H, Ni*x*‐H, and Co*x*‐H systems (Figure 1f). Because the identical reactants are involved in the inverse disproportionation, the free energy differences between the products of the two reactions (Δ*F'*) serve as a proxy of thermodynamic competition. By definition, a more negative value of Δ*F* indicates a stronger tendency of a thermodynamic driving force. All three elements exhibit a positive effect, with the substitution of Cu, Ni, and Co increasing the Δ*F'* from the original −195.06 kJ mol^−1^ to −37.68, −45.31, and −89.06 kJ mol^−1^, respectively. The details of the calculation can be found in Table S1 (Supporting Information). This suggests a greater inclination toward Reaction (2) with enhanced thermodynamic competitiveness by substituting Cu and Ni. The free energy variation exhibits a trend similar to the decreased formation energy, which indirectly demonstrates that the Co/Ni/Cu substitution thermodynamically favors Zr_2_Fe over Zr_3_Fe.

**Figure 2:**
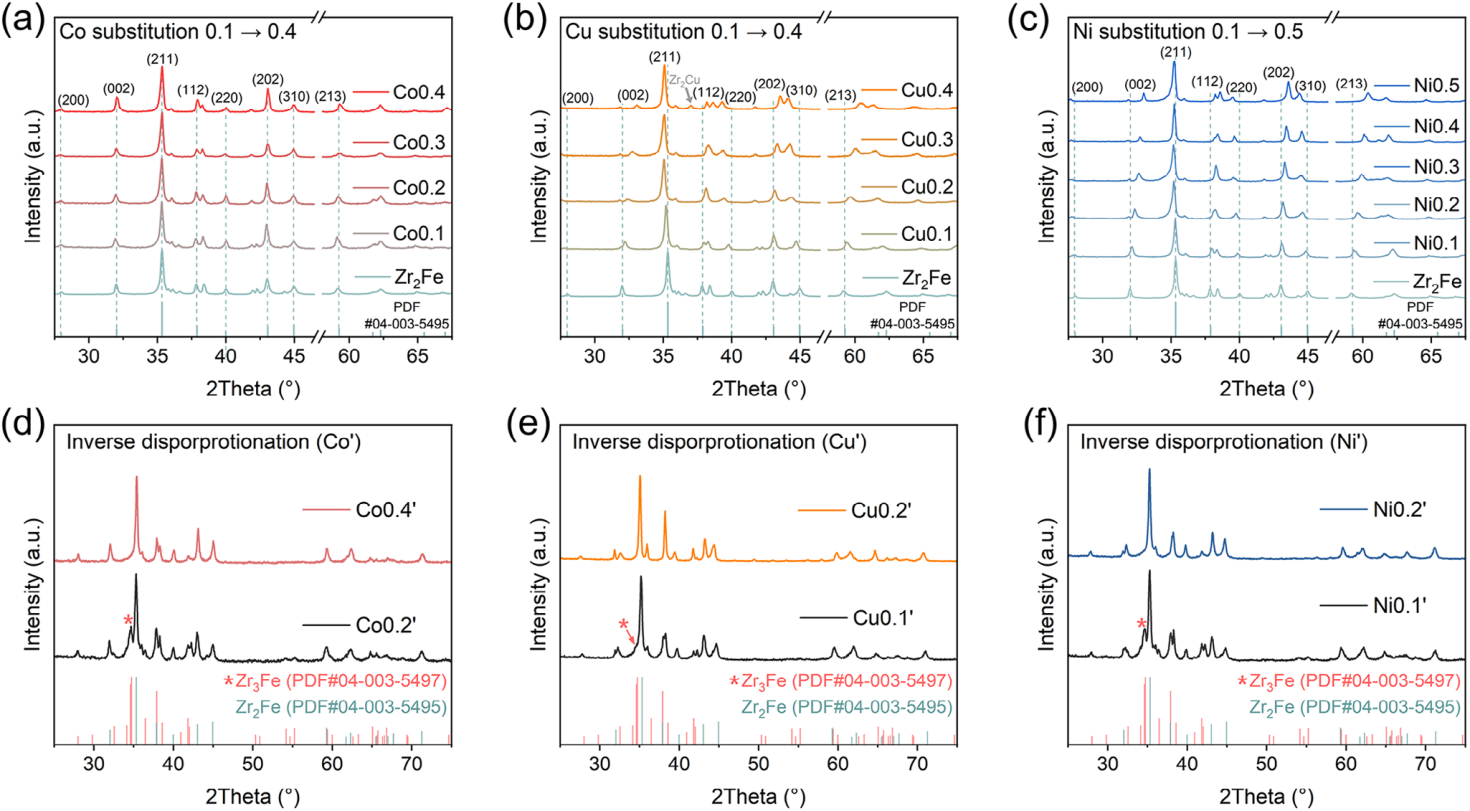
XRD patterns of the as‐cast Zr_2_Fe_1‐_ *_x_* Co*_x_* (*x* = 0–0.4) (a), Zr_2_Fe_1‐_ *_x_* Cu*_x_* (*x* = 0–0.4) (b) and Zr_2_Fe_1‐_ *_x_* Ni*_x_* (*x* = 0–0.5) (c) alloys; XRD patterns of the inverse disproportionation products of Zr_2_Fe_1‐_ *_x_* Co*_x_* (*x* = 0.2, 0.4) (d), Zr_2_Fe_1‐_ *_x_* Cu*_x_* (*x* = 0.1, 0.2) (e) and Zr_2_Fe_1‐_ *_x_* Ni*_x_* (*x* = 0.1, 0.2) (f).

Accordingly, alloys with atomic substitution not exceeding 0.5 were prepared with consideration of solid solubility in the Zr_2_Fe matrix. The scanning electron microscopy (SEM) images along with the energy‐dispersive X‐ray spectroscopy (EDS) results of the Zr_2_Fe_1‐_ *_x_* M*_x_* alloys are exhibited in Figure S2 and Table S2 (Supporting Information), which demonstrate effective alloying and homogeneous elemental distribution. From corresponding XRD results (**Figure** **2**a–c), it is evident that all the alloys maintained the body‐centered tetragonal structure. The segregated phases ZrFe_2_ and Zr are suppressed with substitution, initially suggesting the stabilizing effect of alloying of Co, Cu, and Ni. As shown in Figure 2a, Co substitution for Fe has minimal impact on the crystal structure of Zr_2_Fe due to the similar atomic sizes and electronic configurations (3*d^6^* 4*s^2^* for Fe and 3*d^7^* 4*s^2^* for Co). The diffraction peaks exhibit significant shifts following the substitution of Cu and Ni (Figure 2b–c), with those at 28° (200), 35.3° (211), 40°(220) and 45° (310) shifting toward lower angles, while the peaks at 32° (002), 37.9° (112), 43° (202) and 59.26° (213) shift toward higher angles. In opposing peak shifts, the diffraction peaks corresponding to (*hkl*) planes with *l* = 0 generally move to lower angles. According to Bragg's law (2*d*·sin*θ* = *nλ*) and the tetragonal interplanar spacing equation ($\frac{1}{{d^{2}}_{hkl}}=\;\frac{h^{2}+k^{2}}{a^{2}}+\frac{l^{2}}{c^{2}}$), this trend indicates lattice expansion along the *a*/*b* axes. In contrast, diffraction peaks of planes with *l* ≠ 0 shift toward higher angles, suggesting contraction along the *c*‐axis. Particularly, the (211) peak, despite having *l* ≠ 0, instead exhibits a slight shift toward lower angles, which is attributed to a markedly increased a/c ratio, indicative of pronounced lattice distortion. This distortion is ascribed to inhomogeneous crystallographic stress resulting from the significant differences in atomic radius, electronegativity, and electronic configurations between Fe (3*d^6^* 4*s^2^*) and the substituting elements Ni (3*d^8^* 4*s^2^*) and Cu (3*d^10^* 4*s^1^*). The corresponding lattice parameter information was calculated to investigate the relatively large variations in Cu*x* and Ni*x* alloys, as listed in **Table** **1**. The lattice constants *a* and *b* increase monotonically with Cu and Ni substitution, while *c* decreases monotonically, leading to a continuous increase in the *a*/*c* ratio. Lattice distortion along the *a*/*b* axes becomes less pronounced at higher substitution concentrations, and the cell volume reaches the maximum when *a*/*c* is ≈1.175 (V_Cu0.2_ = 230.04 Å^3^, V_Ni0.3_ = 227.78 Å^3^), followed by shrinkage due to further substitution by smaller‐radius atoms. It is shown that Cu and Ni substitutions induce a similar distortion pattern in the Zr_2_Fe alloy, with Cu causing more pronounced distortion due to its greater atomic property differences compared to Fe. Furthermore, when the Cu substitution reaches 0.4, a distinct Zr_2_Cu segregation phase appears (Figure 2b), and EDS line scanning of the Cu0.4 sample reveals multiple distinct regions enriched in Cu and Fe within the alloy (Figure S3, Supporting Information), indicating that the Cu content exceeds its solid solubility limit in Zr_2_Fe at this composition.

**Table 1:** Lattice parameters of Zr_2_Fe_1‐_ *_x_* Cu*_x_* (*x* = 0–0.5) and Zr_2_Fe_1‐_ *_x_* Ni*_x_* (*x* = 0–0.5) alloys.

Alloy	Lattice parameters [Å]			Volume [Å^3^]	*a*/*c*
	*a*	*b*	*c*		
Zr_2_Fe	6.369	6.369	5.591	226.771	1.139
Cu0.1	6.407	6.407	5.558	228.121	1.153
Cu0.2	6.467	6.467	5.500	230.041	1.176
Cu0.3	6.475	6.475	5.454	228.684	1.187
Cu0.4	6.478	6.478	5.426	227.730	1.194
Ni0.1	6.391	6.391	5.563	227.193	1.149
Ni0.2	6.413	6.413	5.531	227.492	1.160
Ni0.3	6.444	6.444	5.485	227.777	1.175
Ni0.4	6.445	6.445	5.457	226.714	1.181
Ni0.5	6.448	6.448	5.428	225.665	1.188

Subsequently, the above alloys were subjected to the process of inverse disproportionation dehydrogenation, and the XRD patterns of the products are exhibited in Figure 2d–f. Substituting Co for 0.2 atomic weight of Fe can partially suppress the formation of Zr_3_Fe during the inverse disproportionation reaction, and it converts the inverse disproportionation process from Reaction (1) to Reaction (2) until the Co substitution reaches 0.4 (Figure 2d). Cu substitution proves more effective, as Cu0.1 largely improves the thermodynamic favorability of the Zr_2_Fe phase, while Cu0.2 enables a fully regular disproportionation reaction (Figure 2e). The effect of Ni substitution is slightly weaker than that of Cu, but at an atomic substitution of 0.2, it also achieves comparable reversibility (Figure 2f). Figure S4 (Supporting Information) compares the initial hydrogenation kinetics with those after inverse disproportionation treatment for Zr_2_Fe and the reversible Cu0.2 and Ni0.2 alloys, as well as the XRD patterns of their corresponding final hydrides. It can be observed that the hydrogen storage performance of Zr_2_Fe is severely destroyed due to distorted inverse disproportionation. In contrast, Cu0.2 and Ni0.2 maintain their original excellent performance. These results demonstrate that Co, Cu, and Ni substitutions‐all can shift the thermodynamic preference during inverse disproportionation and achieve full reversibility, with the effectiveness in the order of Cu>Ni>Co, in agreement with theoretical calculations as shown in Figure 1.

The improvement of the hydriding disproportionation resistance of Zr_2_Fe alloys is the key to enhancing the hydrogen storage cycling performance, and in our previous studies, it was learned that the hydriding disproportionation reaction in the Zr_2_Fe‐H system is mainly induced by high temperatures and largely influenced by the kinetics, with the reaction equation:

(3) $$Zr_{2}\mathrm{Fe}H_{x}+H_{2}\to \;\mathrm{Zr}H_{2}+\;\mathrm{ZrF}e_{2}$$

**Figure 3:**
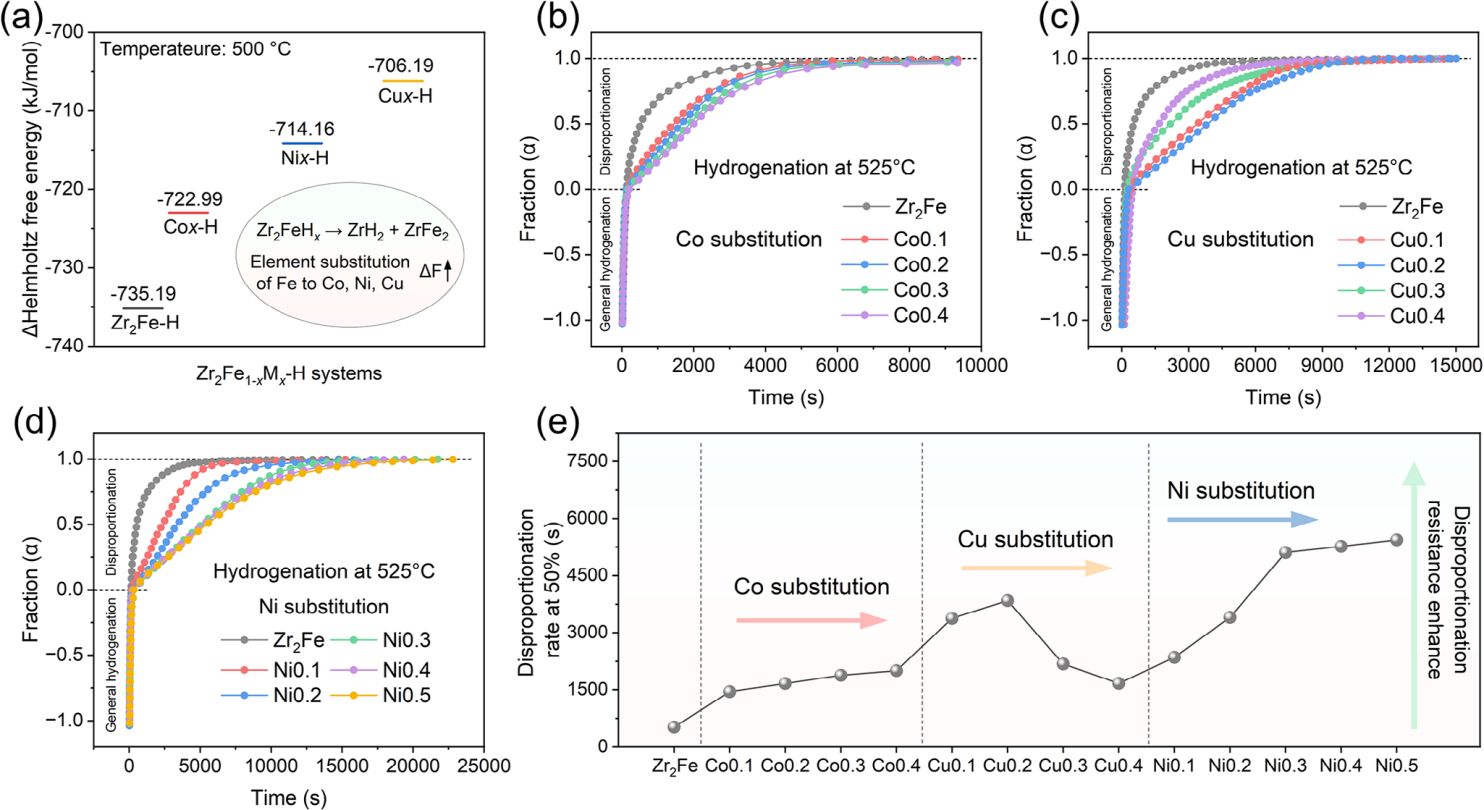
Helmholtz free energy calculations for the hydriding disproportionation reactions at 500 °C in Zr_2_Fe_1‐_ *_x_* M*_x_*‐H systems (a); Normalized hydrogenation kinetics curves of Zr_2_Fe_1‐_ *_x_* Co*_x_* (b), Zr_2_Fe_1‐_ *_x_* Cu*_x_* (c) and Zr_2_Fe_1‐_ *_x_* Ni*_x_* alloys (d) under a hydrogen pressure of 0.05 bar at 525 °C; The comparison of the time for disproportionation progress to 50% in Zr_2_Fe‐based alloys with different element substitutions (e).

The stability of the hydrogen storage system is a key factor affecting the driving force of the hydriding disproportionation reaction. Therefore, we further explored the effect of the three stabilization‐propensity alloying elements (Co, Cu, and Ni) on the disproportionation resistance in Zr_2_Fe_1‐_ *_x_* M*_x_*‐H systems. Helmholtz free energy calculations were again employed to preliminarily assess the thermodynamic difficulty of the disproportionation reaction at 500 °C (**Figure** **3**a). The results show that the Co*x*‐H, Cu*x*‐H, and Ni*x*‐H systems increase the Δ*F* of the hydriding disproportionation reaction (Reaction 3) from −735.19 kJ mol^−1^ for the Zr_2_Fe‐H system to −722.99, −714.16, and −706.19 kJ mol^−1^, respectively. This indicates that all three alloying elements can reduce the thermodynamic feasibility of the hydriding disproportionation reaction in the Zr_2_Fe‐H system, with effectiveness following the order of Cu > Ni > Co.

Next, hydrogen absorption experiments at 525 °C were conducted to assess the impact of substituting different amounts of Co, Cu, and Ni for the Fe on the anti‐disproportionation performance of the Zr_2_Fe‐H system (Figure 3b–d). Since the hydrogen storage capacity and equilibrium pressure of the substituted Zr_2_Fe_1‐_ *_x_* M*_x_* alloys have slight changes, the hydrogen absorption reactions are normalized to better contrast the progression of the disproportionation. General hydrogen absorption and disproportionation hydrogen absorption can be distinctly divided due to the markedly different kinetics, with the fraction (*α*) from ‐1 to 0 representing the general hydrogenation reaction progress, and from 0 to 1 representing the disproportionation reaction progress. The disproportionation kinetics curves of these alloys show considerable differences, allowing for a clear comparison of their resistance to disproportionation. Figure 3e summarizes the time required for the disproportionation progress fraction (*α*) to reach 0.5 for each alloy. It is observed that Co substitution offers limited improvement in the disproportionation resistance of Zr_2_Fe, whereas Cu and Ni substitutions result in markedly enhanced modification effects. With increasing Cu substitution, the time for 50% disproportionation increases from 524 s for Zr_2_Fe to 3846 s for Cu0.2, indicating a substantial improvement. However, Cu0.3 and Cu0.4 alloys exhibit a decline in disproportionation resistance, deviating from the expected trend. This is attributed to the excessive Cu inducing segregation of the Zr_2_Cu, which has poor disproportionation resistance. Moreover, segregated phases are known to facilitate the disproportionation reaction.^[^ ^23^, ^24^, ^25^ ^]^ The disproportionation resistance of Ni0.1 and Ni0.2 alloys is slightly weaker than that of Cu0.1 and Cu0.2, respectively, which is in accordance with the analysis of the Helmholtz free energy calculations for Reaction (3). Notably, the Ni0.3 does not show a decline in disproportionation resistance like the Cu*x* alloys, instead further prolonging the time to reach 50% disproportionation to 5108 s. However, further Ni substitution leads to only slight improvements in anti‐disproportionation performance, following a similar incremental trend as the distortion observed in the crystal structure of modified alloys. The nonlinear relationship between Ni content and disproportionation resistance suggests that the reaction is not only restricted by thermodynamic factors.

**Figure 4:**
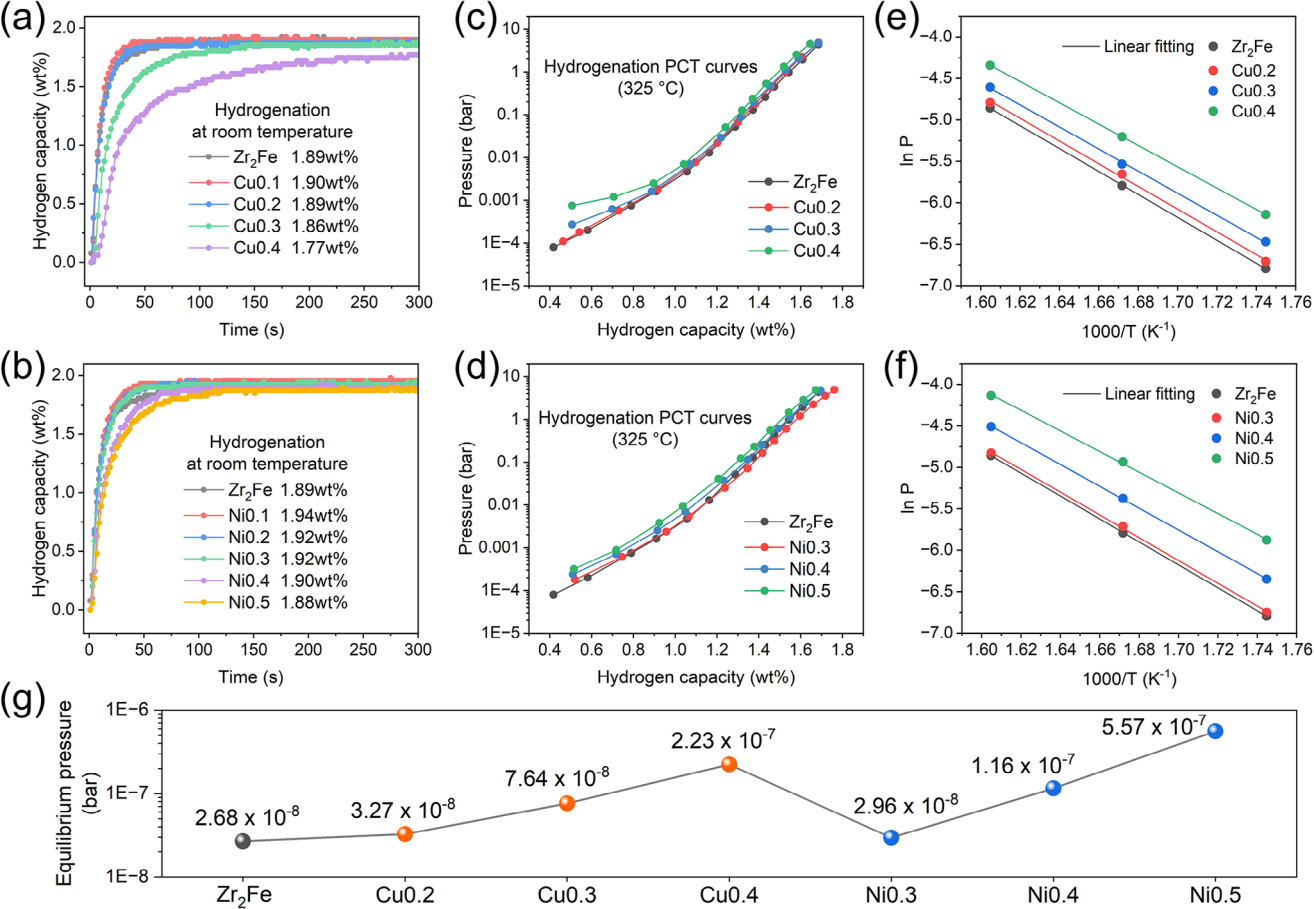
Hydrogenation kinetics under a hydrogen pressure of 0.25 bar at 25 °C (a, b), hydrogenation PCT curves at 325 °C (c, d), and the corresponding Van't Hoff plots (e, f) of Zr_2_Fe, Zr_2_Fe_1‐_ *_x_* Cu*_x_* and Zr_2_Fe_1‐_ *_x_* Ni*_x_* alloys; The comparison of room‐temperature hydrogenation equilibrium pressure of several alloy samples (g).

Hydrogen storage properties are important indicators for assessing the practicality of Cu*x* and Ni*x* alloys with markedly enhanced anti‐disproportionation performance as ultra‐low pressure tritium getter materials. The room‐temperature hydrogenation kinetics curves of the Cu*x* alloys are presented in **Figure** **4**a. Cu0.1 and Cu0.2 maintain relatively rapid kinetics and hydrogen storage capacity comparable to Zr_2_Fe, whereas Cu0.3 and Cu0.4 exhibit significantly slowed kinetics. Figure 4b demonstrates the rapid hydrogenation kinetics of the Ni*x* alloys, where the addition of Ni increases the hydrogen storage capacity to 1.94 wt.% at Ni0.1. Similarly, cell shrinkage leads to a decline in kinetics and capacity, but these effects are less pronounced in the Ni*x* alloys than in the Cu*x* ones at the same unit cell volume, owing to the stronger hydrogen affinity of Ni compared to Cu.^[^ ^26^, ^27^ ^]^ Furthermore, the hydrogenation Pressure‐Composition‐Temperature (PCT) tests were conducted to further examine the effect of alloying on hydrogenation equilibrium pressure. Figure 4c,d presents the hydrogenation PCT curves of Cu*x* and Ni*x* alloys at 325 °C. It is visible that the PCT curves of Cu0.2 and Ni0.3 almost overlap with Zr_2_Fe, and the curve of Ni0.3 broadens because of its increased capacity. Once the crystal cells of the Cu*x* and Ni*x* alloys undergo shrinkage, it is followed by a significant uplift in the PCT curves, representing the rising hydrogenation equilibrium pressure. In addition, there is an upward warping of the PCT curves of Cu*x* alloys at low hydrogen content. This phenomenon can also be attributed to the weaker hydrogen affinity of Cu, further reflecting how the crystal structure and the constituent elements influence the hydrogen storage properties of Zr_2_Fe‐based alloys.^[^ ^28^ ^]^

The hydrogenation PCT curves of Cu*x* and Ni*x* alloys within the temperature range of 300–350 °C are presented in Figure S5 (Supporting Information), and the corresponding specific hydrogenation thermodynamic parameters, including Δ*H* and Δ*S*, are listed in Table S3 (Supporting Information). These are derived from the fitting curves of Van't Hoff plots (Figure 4e,f) according to the equation (ln *P*_eq_ = – Δ*H*/R*T* + Δ*S*/R), where Δ*H* and Δ*S* represent enthalpy and entropy changes of hydrogenation, while R and *T* denote the molar gas constant and the experimental temperature, respectively. Due to the non‐significant plateau characteristic of the PCT curves, the hydrogen pressure corresponding to the midpoint of the theoretical saturation capacity (≈1 wt.%) is adopted as the equilibrium hydrogen pressure (*P*_eq_).^[^ ^29^, ^30^, ^31^, ^32^, ^33^ ^]^ Thus, the room‐temperature hydrogenation equilibrium pressures (25 °C) of the alloys were extrapolated from the Van't Hoff calculations, as illustrated in Figure 4g. It can be found that the absolute values of the Δ*H* are negatively associated with the *P*_eq_ and the equilibrium hydrogen pressures of Zr_2_Fe_1‐_ *_x_* M*_x_* increase exponentially with excessive substitution of Cu and Ni. The Cu0.2 and Ni0.3 alloys largely retain the excellent hydrogen absorption properties as Zr_2_Fe and exhibit ultra‐low hydrogenation equilibrium pressures (25 °C) of 3.27 × 10^−8^ Pa and 2.96 × 10^−8^ Pa, respectively, ensuring the efficient recovery of tritium at extremely low concentrations. Consequently, they were chosen as the preferred Zr_2_Fe_1‐_ *_x_* M*_x_* alloys, owing to their excellent hydrogen absorption properties and significantly enhanced disproportionation resistance.

**Figure 5:**
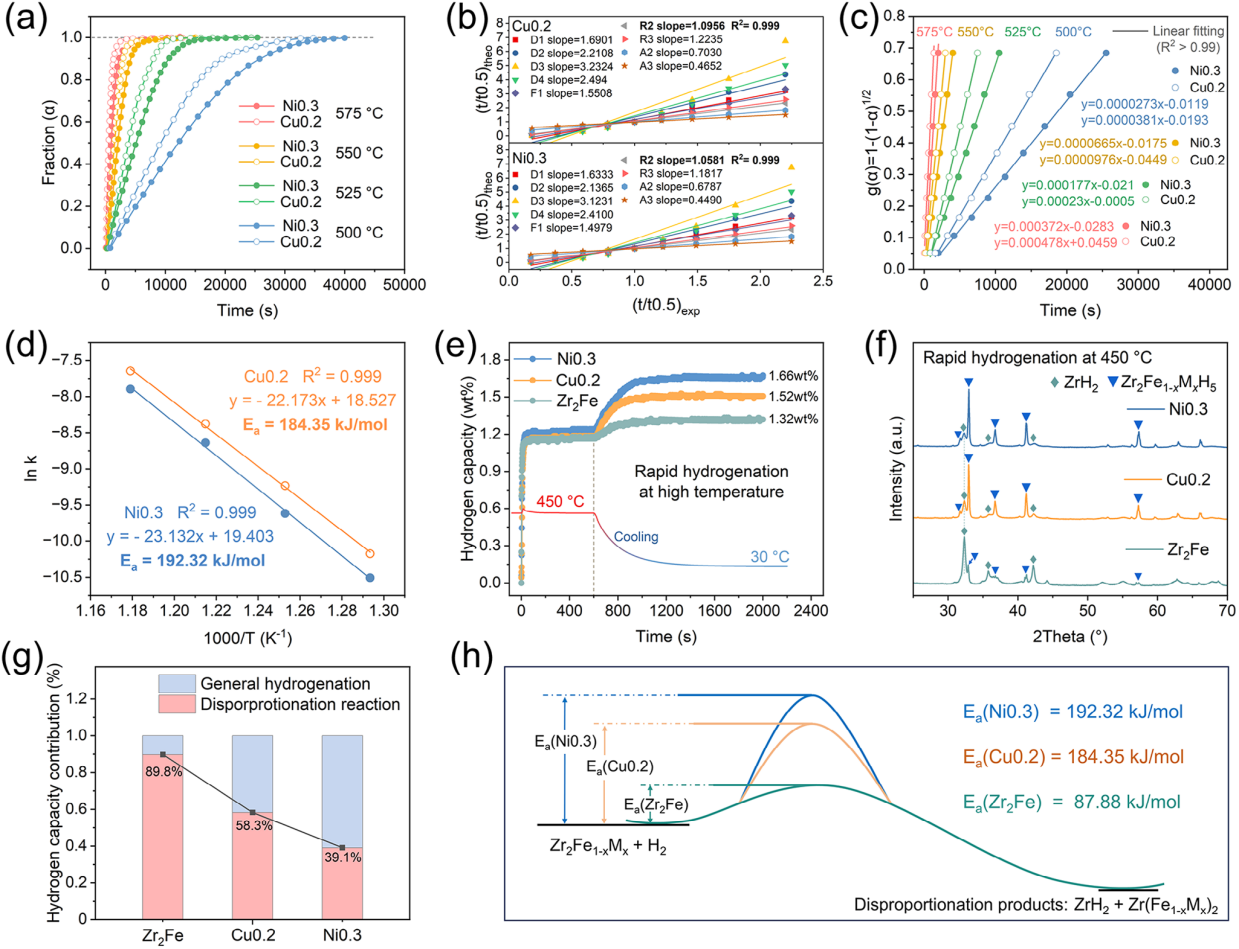
Normalized kinetics curves of hydriding disproportionation reaction of Zr_2_Fe_0.8_Cu_0.2_ and Zr_2_Fe_0.7_Ni_0.3_ alloys under hydrogen pressure of 0.05 bar at 500, 525, 550, 575 °C (a); (t/t_0.5_)_theo_ versus (t/t_0.5_)_exp_ of Zr_2_Fe_0.8_Cu_0.2_ and Zr_2_Fe_0.7_Ni_0.3_ alloys for various kinetics models (b); Time dependence of R2 kinetics modeling equations g(*α*) for Zr_2_Fe_0.8_Cu_0.2_ and Zr_2_Fe_0.7_Ni_0.3_ alloys (c); Activation energy calculation using Arrhenius plot (d); Hydrogenation kinetics curves of Zr_2_Fe, Zr_2_Fe_0.8_Cu_0.2_ and Zr_2_Fe_0.7_Ni_0.3_ alloys under hydrogen pressure of 1.0 bar at 450 °C (e) and the XRD patterns of corresponding products (f), and the hydrogen capacity contribution from the general hydrogenation reaction and hydriding disproportionation reaction (g); The schematic diagram of apparent activation energy for hydriding disproportionation reaction (h).

For the Cu0.2‐H and Ni0.3‐H systems with outstanding overall performance, further investigation was conducted on the decelerated disproportionation kinetics, with the apparent activation energy (*E*_a_) initially introduced to evaluate changes in the energy barrier. **Figure** **5**a exhibits the kinetics curves for the hydriding disproportionation reaction of Cu0.2 and Ni0.3 alloys over the temperature range of 500–575 °C. The disproportionation kinetics of Ni0.3 are consistently slower than those of Cu0.2 at the same temperature. Subsequently, a practical approach was adopted to identify the kinetic model for the disproportionation reaction, with the specific derivation method detailed in previous studies.^[^ ^5^, ^34^, ^35^ ^]^ Figure 5b presents the plots of the experimental values (*t*/*t*_0.5_)_exp_ versus the modeled theoretical values (*t*/*t*_0.5_)_theo_, where *t_α_* denotes the time corresponding to the reaction progress fraction *α*. The 2D phase boundary model (R2) achieves a fitting slope value of 1.09561 and 1.05811 for Cu0.2 and Ni0.3, respectively, with correlation coefficients (R^2^) of 0.999 in both cases. The good linear relationship indicates that the disproportionation reaction of Cu0.2 and Ni0.3 can be reasonably interpreted by the R2 model. In contrast, the kinetic model for Zr_2_Fe is identified as 3D phase boundary model (R3) in our previous work. Both the R2 and R3 models are classified as shrinking core models, which describe reactions that initially occur on the outer surface of a particle and gradually progress inward, leaving the unreacted solid as a shrinking core.^[^ ^36^, ^37^, ^38^ ^]^ This indicates that Cu and Ni modifications of Zr_2_Fe follow a consistent mechanism regulating disproportionation kinetics, and the shift from R3 to R2 in the kinetic model may reflect the inhibition of the geometrical extension of ZrH_2_ and ZrFe_2_. Subsequently, the excellent fitting of the experimental reaction at various temperatures to the model formula (R2, g(*α*) = 1‐(1‐*α*)^1/2^) further confirms the high compatibility of the selected kinetic model (Figure 5c). Based on this, the activation energy (*E*_a_) can be calculated using the Arrhenius equation, *k*(*T*) = A · exp(–*E*_a_/R*T*), where A, R, *T*, and *k*(*T*) denotes the pre‐exponential factor, the molar gas constant, the test temperature and the reaction rate coefficient that derived from Figure 5c, respectively. The *E*_a_ values for the hydriding disproportionation reaction of Cu0.2 and Ni0.3 were calculated as 184.35 and 192.32 kJ mol^−1^, respectively, exhibiting a significant increase compared to 87.88 kJ mol^−1^ of Zr_2_Fe alloy (Figure 5d).

The kinetic inhibition of the disproportionation reaction in Cu0.2‐H and Ni0.3‐H systems was further demonstrated during the high‐temperature rapid hydrogenation process. As shown in Figure 5e, at 450 °C, all alloys underwent a rapid hydrogen absorption reaction due to the relatively high hydrogen pressure (1 bar), involving both general hydrogenation and hydriding disproportionation. During the subsequent cooling process, the hydrides of Zr_2_Fe_1‐_ *_x_* M*_x_* could be hydrogenated to saturation, whereas the disproportionation products were incapable of further hydrogen uptake, exposing the detrimental impact of disproportionation on hydrogen storage performance. The final retained hydrogen storage capacities of Ni0.3, Cu0.2, and Zr_2_Fe are 1.66, 1.52, and 1.32 wt.%, respectively. Figure 5f shows the XRD patterns of the corresponding products, where the diffraction peaks of the disproportionation product ZrH_2_ are noticeably weakened in the Cu0.2 and Ni0.3 after rapid hydrogenation at 450 °C. Based on the hydrogen requirement of 1.26 wt.% for the complete disproportionation reaction of Zr_2_Fe, the respective contributions to the hydrogen storage capacity of the general hydrogenation reaction and the disproportionation reaction to the overall capacity were calculated, as shown in Figure 5g. The disproportionation rate decreases sharply from 89.8% for Zr_2_Fe to 39.1% for Ni0.3. It suggests that Ni0.3 and Cu0.2 possess considerably improved resistance to lattice disturbances induced by intense atomic migration, thereby substantially suppressing the kinetics of the disproportionation reaction. Furthermore, Figure 5h depicts the kinetic inhibition of the hydrogen absorption disproportionation reaction by high activation energy barriers in the Ni0.3‐H and Cu0.2‐H systems, with Ni0.3 being the most effective.

**Figure 6:**
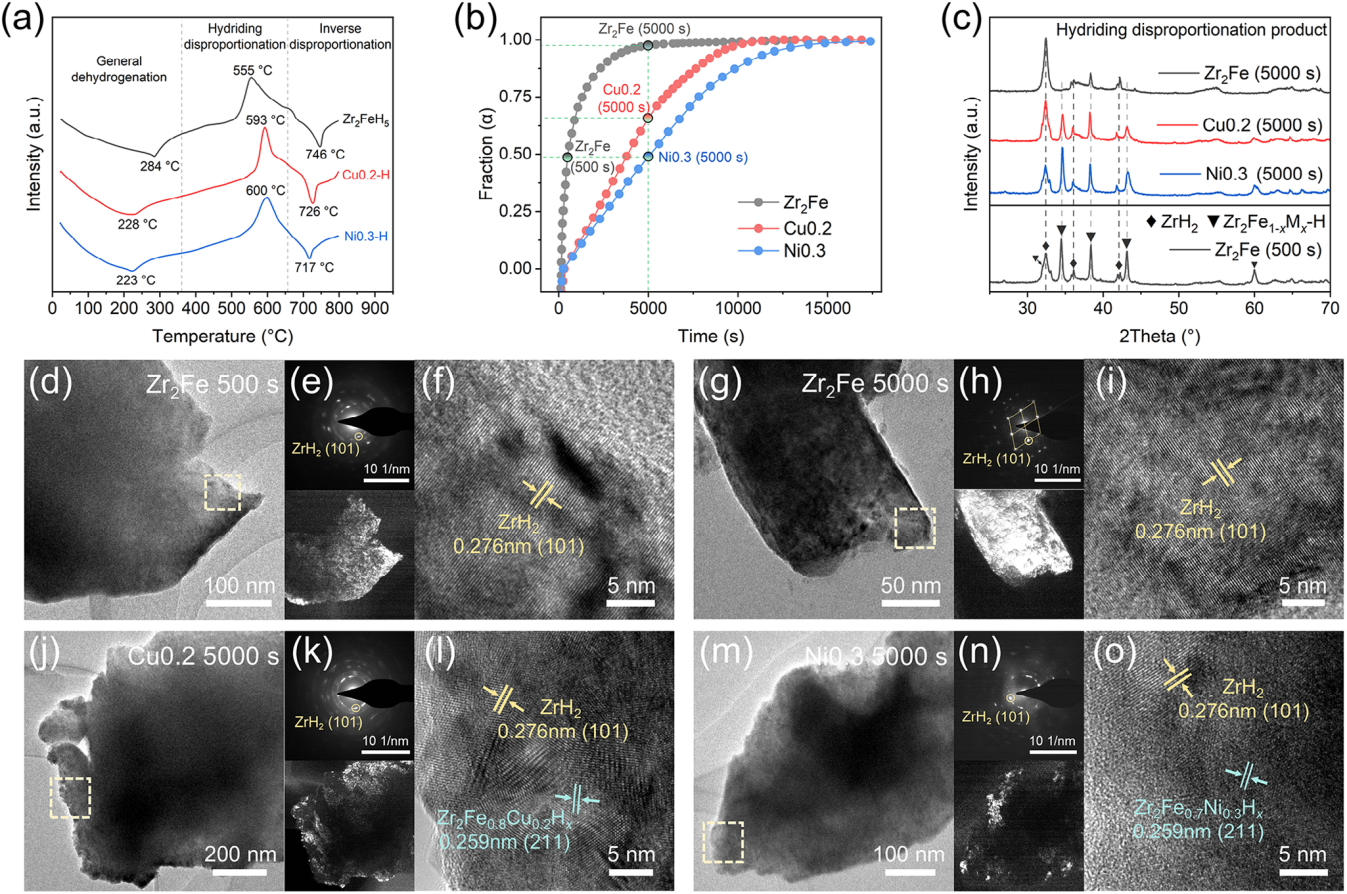
DSC curves of Zr_2_FeH_5_, Zr_2_Fe_0.8_Cu_0.2_H_5_ and Zr_2_Fe_0.8_Ni_0.3_H_5_ hydrides at a heating rate of 10 °C min^−1^ (a); Hydriding disproportionation kinetics curves at 525 °C of Zr_2_Fe, Zr_2_Fe_0.8_Cu_0.2_ and Zr_2_Fe_0.7_Ni_0.3_ alloys (b), and the corresponding XRD patterns (c); TEM images for the marked points in kinetics curves (d, g, j, m), and the SAED photographs with centered dark‐field TEM images acquired using the ZrH_2_ phase reflection marked by yellow cycle in SAED photographs (e, h, k, n), and the corresponding HRTEM images at dashed box selection (f, i, l, o).

The kinetic thermodynamic constraints on the disproportionation reaction can also be explicitly demonstrated through differential scanning calorimetry (DSC) tests (**Figure** **6**a). The significant shift of the exothermic peak of the hydriding disproportionation reaction toward higher temperatures is observed, increasing from 555 °C in the pristine Zr_2_FeH_5_ to 593 and 600 °C in the Cu0.2‐H and N0.3‐H, respectively. Correspondingly, the apparent activation energy of hydriding disproportionation under DSC test condition (*E*_a_‐DSC) increases significantly from 106.75 kJ mol^−1^ in the Zr_2_Fe‐H system to 245.35 and 265.47 kJ mol^−1^ in the Cu0.2‐H and Ni0.3‐H systems, respectively (Figure S6, Supporting Information), in good agreement with the above discussion on energy barrier analysis in high‐temperature hydrogenation. The exothermic peak areas are markedly reduced after alloying modification, with the enthalpy changes decreasing from −20.90 kJ mol^−1^ (Zr_2_FeH_5_) to −12.02 kJ mol^−1^ (Cu0.2‐H) and −10.23 kJ mol^−1^ (Ni0.3‐H), corresponding to the increasing trend of Δ*F* illustrated in Figure 3a, indicating a reduced thermodynamic driving force. Furthermore, elemental substitution leads to a lower decomposition temperature of the disproportionation products (inverse disproportionation), reflecting reduced chemical stability of the disproportionation phase Zr(Fe_1‐_ *_x_* M*_x_*)_2_. The experimental result correlates well with the elevated free energy (*F*) of the substituted Zr(Fe_1‐_ *_x_* M*_x_*)_2_ at 700 °C (Figure S7, Supporting Information). These findings collectively provide evidence of the suppression of the disproportionation reaction through both kinetic and thermodynamic modulation.^[^ ^39^, ^40^, ^41^, ^42^ ^]^ Moreover, it is worth mentioning that general dehydrogenation is more accessible in the preferred system, and the lower operating temperatures for both general dehydrogenation and inverse disproportionation reactions are greatly beneficial to the application.

To acquire a more comprehensive understanding of the disproportionation behaviors and the modification effects, further characterization of the phase composition and distribution during the reaction was carried out. As shown in Figure 6b, under the same hydrogen environment, Zr_2_FeH*_x_* hydride spent 500 s being disproportionated to 50% at 525 °C, and the disproportionation reaction was almost complete within 5000 s. In contrast, Zr_2_Fe_0.8_Cu_0.2_H*_x_* and Zr_2_Fe_0.7_Ni_0.3_H*_x_* were disproportionated to ≈50% and 65% at 5000 s, respectively. The XRD analyses of the intermediate states are exhibited in Figure 6c, revealing that the transition of hydride to disproportionation products in the Ni0.3‐H system is relatively the most strongly inhibited. Furthermore, transmission electron microscopy (TEM) microscopic characterization was performed to better reflect the invasion process of disproportionation reaction in the Cu*x*‐H and Ni*x*‐H hydrides. In particular, centered dark‐field TEM images, obtained using the reflection of the ZrH_2_ phase marked by a yellow circle in the selected area electron diffraction (SAED) pattern, were utilized to clearly visualize the distribution of the disproportionated phase within the particle, where the bright regions correspond to the distribution of ZrH_2_ phase.^[^ ^43^ ^]^ Figure 6d presents the TEM image of the Zr_2_Fe 500 s point shown in Figure 6b, with the corresponding SAED diffraction spots showing a polycrystalline pattern (Figure 6e). High‐resolution TEM (HRTEM) observations of these bright areas reveal continuous lattice fringes corresponding to ZrH_2_, while the lattice fringes of the hydride phase are difficult to discern (Figure 6f). At the late stage of disproportionation (5000 s), the SAED pattern clearly shows the diffraction spots associated with the body‐centered tetragonal structure, and the corresponding dark‐field image shows an almost full distribution of the ZrH_2_ phase in the particle (Figure 6g,h). This indicates a rapid process of growth and coalescence of highly oriented ZrH_2_ grains during the disproportionation in the Zr_2_Fe‐H system, which corresponds to the progressively narrowing ZrH_2_ diffraction peaks in Figure 6c. The above results are exemplified by the large‐scale extension of the lattice fringes of ZrH_2_ (101) in the HRTEM observation (Figure 6i). During the disproportionation in Cu0.2‐H and Ni0.3‐H systems, the ZrH_2_ phase exhibits a scattered and edge‐localized distribution (Figure 6k,n). The HRTEM images corresponding to the yellow boxes in Figure 6j and m both show polycrystalline fringes, including lattice fringes of ZrH_2_ and those of Zr_2_F_e0.8_Cu_0.2_H*_x_* and Zr_2_Fe_0.8_Ni_0.3_H*_x_*, respectively, where the lattice fringes associated with the hydride phase are observed on the inner side (Figure 6l,o). The suppression of ZrH_2_ grain growth is more pronounced in the Ni0.3‐H system, indicating a relatively better disproportionation resistance. This reflects the significantly inhibited 3D inward extension kinetics of the disproportionation phase within the hydride structure, as supported by the kinetic analyses discussed above.

**Figure 7:**
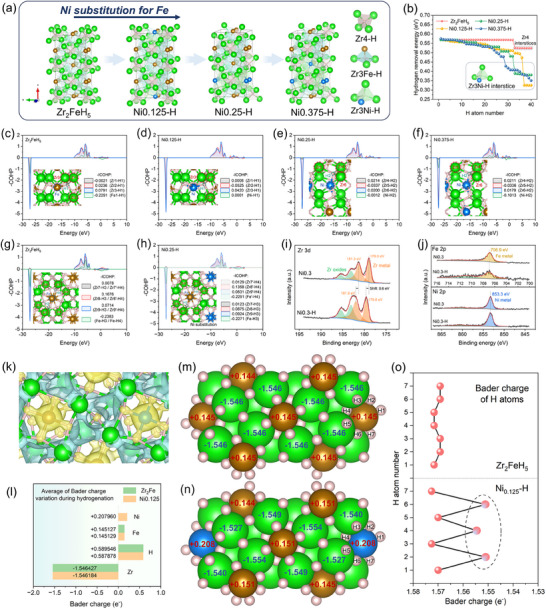
Optimized supercell structures of Zr_2_Fe_1‐_ *_x_* Ni*_x_* H_5_ (*x* = 0, 0.125, 0.25, 0.375) with Zr4‐H, Zr3Fe‐H and Zr3Ni‐H interstices (a); Hydrogen removal energies at different interstices in Zr_2_Fe_1‐_ *_x_* Ni*_x_*‐H (*x* = 0, 0.125, 0.25, 0.375) systems (b); The Zr3Fe/Ni‐H interstices and calculated COHP between Zr, Fe/Ni and H atom in Zr_2_FeH_5_ (c), Ni0.125‐H (d), Ni0.25‐H (e) and Ni0.375‐H (f); The Zr3Fe‐H interstices and calculated COHP between Zr, Fe and H atom in Zr_2_FeH_5_ (g) and Ni0.25‐H (h); Zr 3d (i), Ni 2p and Fe 2p (j) XPS spectrum of Zr_2_Fe_0.7_Ni_0.3_ and Zr_2_Fe_0.7_Ni_0.3_H_5_; The differential charge density diagram in Zr_2_Fe_0.75_Ni_0.125_H_5_ structure, where in the charge accumulation region is rendered in yellow, and the charge depletion is in blue (k); Bader charge variations during hydrogenation in Zr_2_Fe‐H and Ni0.125‐H systems (l); Bader charge distribution in the Zr_2_FeH_5_ (m) and Ni0.125‐H (n); Bader charge values of specific H atoms in Zr_2_FeH_5_ and Ni0.125‐H (o).

For a deeper understanding, the inhibition mechanism of the disproportionation reaction is further analyzed by combining DFT calculations. First, the supercell structures of the hydrides of Zr_2_Fe, Ni0.125, Ni0.25, and Ni0.375 are optimized (**Figure** **7**a). The effect of the substituted atoms on Metal‐H interaction strength at hydrogen storage interstices is evaluated via hydrogen binding energy calculations, as shown in Figure 7b for the Ni*x*‐H systems. Following Ni substitution, hydrogen removal energies of the various hydrogen storage interstices decrease and become continuous, which facilitates the occurrence of the dehydrogenation reaction.^[^ ^44^, ^45^ ^]^ This result corresponds well to the decrease in dehydrogenation temperature in the DSC analysis (Figure 6a). In addition, the hydrogen removal energies of the Zr3Ni‐H interstices are calculated to be less than 0.45 eV, which is lower than that of the Zr3Fe and Zr4 interstices. The variations in hydrogen removal energies for the Cu*x*‐H systems behave similarly (Figure S8, Supporting Information).

Inspired by hydrogen binding energy variations, the crystal‐orbital Hamilton population (COHP) analysis was introduced to investigate the energy contribution of bonding electrons shared between metal and H atoms. The average integrated COHP (ICOHP) was used to precisely quantify the Metal─H bonding strength. In the substitution progression toward Ni0.125‐H system, when one of the Zr3Fe‐H interstices in Zr_2_FeH_5_ was replaced by a Zr3Ni‐H interstice, the calculated ‐ICOHP values crossing the Fermi level for Zr1─H1, Zr2─H1, Zr3─H1, and Fe─H1 bonds changed from −0.0021, 0.0236, 0.0791 and −0.2291 to 0.0008 (Zr1─H1), ‐0.0525 (Zr2─H1), 0.420 (Zr3─H1) and 0.0001(Ni─H1), respectively (Figure 7c,d). According to the definition, a more negative ICOHP value signifies stronger bonding. It can be obtained that the strength of the Zr3─H1 bond is significantly weakened, particularly with Zr2─H1 adopting an antibonding state after substitution. Further weakening of Zr─H bonds in the Zr3Ni‐H interstice was induced by increased substitutions of Fe with Ni (Ni0.25‐H → Ni0.375‐H), as reflected in the decrease of the ‐ICOHP values for Zr4─H2 and Zr6─H2 bonds from 0.214 and 0.0200 to 0.0211 and 0.0179, respectively (Figure 7e,f). This suggests that under low pressure and high temperature, H atoms in the Zr3Ni‐H interstices are preferentially detached, enabling the non‐bonded metal atoms to actively participate in the hydriding disproportionation reaction, thereby inhibiting the formation of the disproportionation product Zr(Fe_1‐_ *_x_* Ni*_x_*)_2_ due to its greater thermodynamic instability. Nevertheless, experimental results demonstrated that the disproportionation resistance of Ni0.5 and Ni0.4 is marginally improved compared to that of Ni0.3. The existence of an upper limit to the enhancement provided by Ni substitution further demonstrates that it results not only from the thermodynamic suppression but also from the considerable modulation of the electronic structure in Ni*x*‐H systems, thereby synergistically optimizing the resistance to disproportionation.

On the other hand, during the transformation from hydride to disproportionation, it is inevitable for the breaking of Metal─H bonds in the un‐dehydrogenated Zr3Fe‐H interstices. Affected by Ni substitution, the Metal─H bonding strength within the Zr3Fe‐H interstice surrounding the substituted atoms exhibits unusual variations (Figure 7g,h). Specifically, for a pair of Zr3Fe‐H interstices, the ‐ICOHP values for Zr7─H3, Zr8─H3, Zr9─H3 and Fe─H3 bonds changed from 0.0078, 0.1676, 0.0714 and ‐0.2383 to 0.0123, 0.0875, 0.0924 and ‐0.2271, respectively, while the ‐ICOHP values corresponding to Zr7'‐H4, Zr8'‐H4, Zr9'‐H4 and Fe─H4 bonds changed to 0.0123, 0.0875, 0.0924 and ‐0.2271, respectively. It is evident that the strengths of the weakest Zr7‐H3(Zr7'‐H4) bonds are enhanced while those of the strongest Zr8‐H3(Zr8'‐H4) bonds are weakened, and Zr9‐H3(Zr9'‐H4) bonding strengths are synchronously modulated. The standard deviation of Zr─H bond strength within Zr3Fe‐H3 and Zr3Fe‐H4 interstices decreased from 0.0657 to 0.0367 and 0.0503, respectively, exhibiting convergence toward Zr─H bonding strength homogeneity. We also performed this analysis for the Cu*x*‐H system and found a similar trend in bonding strength variations (Figure S9, Supporting Information). This suggests that the relatively high electronegativity of substitution atoms (Ni/Cu) and the resulting lattice distortion together contribute to the electronic redistribution, affecting the anisotropic strength variations of the Metal─H bonds. As a result, the improvement in anti‐disproportionation performance becomes limited as the substitution level approaches a certain threshold. Consequently, in the optimized hydride systems, the reinforcement of the relatively weak Zr─H bonds reduces their propensity for cleavage under thermal stimulation, thereby hindering a steep increase of atomic participation in the initial stage of the disproportionation reaction. The uniform dissociation of stronger Zr─H bonds mitigates the deviation from the ratio of Zr to Fe (2:1), to some extent avoiding the inhomogeneous chemical environment and further reducing the driving force for disproportionation.

As detected by X‐ray photoelectron spectroscopy (XPS) analysis for the Ni*x*‐H system, the Zr 3d binding energy of Zr metal increased by 0.6 eV during hydrogenation, indicating electron delocalization around Zr in the hydrogenation process and confirming the Zr─H polarized bonding (Figure 7i).^[^ ^46^, ^47^ ^]^ During this process, the peaks of Fe 2p and Ni 2p exhibit no visible shift, which verifies the antibonding Fe‐H and Ni‐H in the above COHP calculations. It is evident that the Fe and Ni atoms mainly contribute to the electron modulation in the Ni*x*‐H system (Figure 7j). The electron migration during hydrogenation of Ni*x* alloy could be significantly reflected by the differential charge density diagram (Figure 7k) and the electron localization function (ELF) diagram (Figure S10, Supporting Information), where the electrons around Zr atoms are delocalized and the covalent electrons exhibit a tendency to localize around H atoms to make them negatively charged. Meanwhile, Fe and Ni atoms attract a small number of electrons in the Ni*x*‐H hydride structure. Subsequently, more detailed electron transfer was investigated by combining Barder charge calculations. As shown in Figure 7l, on average, each Zr atom loses 1.55 eV of electrons, while each H atom gains 0.59 eV of electrons during the hydrogenation of Zr_2_Fe. After Ni substitution, the level of electron loss of Zr atoms remains nearly unchanged, and the number of electrons gained by H atoms slightly decreases, which is possibly influenced by the presence of Ni atoms attracting more electrons. The preservation of Zr‐contributed electron levels aligns with the XPS results comparing the Zr_2_Fe‐H system and the Ni*x*‐H system (Figure S11, Supporting Information). The specific presentation of the local region electronic changes on the (001) crystal surface is shown in Figure 7m,n. Obviously, the charge density around Ni is higher than that of Fe, and then it is found that the charge density of partial H atoms around the substituted atoms is significantly reduced (Figure 7o). Exactly, H2, H4, and H6 all associate with the Ni─H bond corresponding to a decrease in Bader charge values of 0.0182, 0.0169, and 0.0180, respectively. The reduced electron acquisition of H atoms within the Zr3Ni‐H interstices reflects lower electron transfer from Zr to H, resulting in weaker hydrogen binding of Zr3Ni‐H interstices. This phenomenon validates the substantial decrease of hydrogen removal energy and Zr─H bonding energy in the Zr3Ni‐H interstices, as well as confirms the important role of electronegativity changes for electronic redistribution. Upon Ni substitution, the electron loss by Zr atoms during hydrogenation also exhibits variations, including both increases and decreases, which demonstrates a modulated electron transfer between Zr and H atoms in the hydride. The specific electron gain of the H atoms during the hydrogenation of each Zr3Fe interstice was summarized in Table S4 (Supporting Information) for further analysis. Predominantly, the H atoms with relatively more localized electrons experience a decrease in electrons gained after Ni substitution, while those with fewer localized electrons gain more electrons, indicating the general homogenizing effect in electron transfer between Zr and H atoms in Zr3Fe‐H interstices. Simultaneously, in conjunction with the homogenization of the Zr─H bonding strength mentioned above, this further implies a more symmetric distribution of electron density and lower dipole moments within Zr3Fe‐H interstices, thereby reinforcing the stability of Zr_2_Fe_1‐_ *_x_* M*_x_*‐H hydrides.^[^ ^48^, ^49^ ^]^ This inference is further supported by the slightly higher average variation in localized electron density of H atoms in the Zr3Fe‐H interstices of the Ni*x*‐H system (0.571 eV vs 0.570 eV in Zr_2_Fe‐H).

## 3 Conclusion

In this work, an element screening method focusing on lower formation energy was adopted to construct more stable Zr_2_Fe‐based hydrogen storage alloys, and successfully correct the distorted inverse disproportionation reaction and eliminate the occurrence of Zr_3_Fe in the Zr_2_Fe‐H system by the substitution of Fe with Co, Cu, and Ni. The thermodynamic competition involved in the inverse disproportionation reaction is well described by Helmholtz's free energy calculations. Besides, the resistance of Zr_2_Fe alloy to hydriding disproportionation is significantly enhanced under the influence of Cu and Ni substitution, and the thermodynamic favorability of the hydriding disproportionation reaction (Zr_2_FeH*_x_* + H_2_ → ZrH_2_ + ZrFe_2_) is accordingly found to be reduced. Furthermore, through hydrogenation experiments along with data fitting, it is demonstrated that the Cu0.2‐H and Ni0.3‐H systems not only maintain rapid hydrogenation kinetics and ultra‐low equilibrium hydrogen pressures (3.26 × 10^−8^ Pa for Cu0.2 and 2.96 × 10^−8^ Pa for Ni0.3), but also greatly increase the hydriding disproportionation energy barriers to 184.35 and 192.32 kJ mol^−1^, respectively, with corresponding decelerations in disproportionation kinetics, as observed in TEM analysis.

To investigate the modification mechanism of the Zr_2_Fe_1‐_ *_x_* Ni*_x_*‐H system with optimal comprehensive performance, DFT calculations reveal that the H atoms can be readily dissociated from Zr3Ni‐H interstices, leading to the active participation of Ni atoms in the hydriding disproportionation reaction, which reduces disproportionation driving force due to the greater instability of Zr(Fe_1‐_ *_x_* Ni*_x_*)_2_ phase. Simultaneously, by correlating the improved modification performance with variations in Metal─H bonding strength and charge density, the Ni substitution is demonstrated to effectively modulate the electronic redistribution within the hydride system. This strengthens the weak Zr─H bonds and homogenizes the binding energies of the Zr─H bonds in Zr3Fe‐H interstices. As a result, the optimized systems become more stable and less prone to forming chemical environments conducive to disproportionation when exposed to high temperatures, thereby further inhibiting hydriding disproportionation reaction synergistically. A similar modulation is also demonstrated in the Zr_2_Fe_1‐_ *_x_* Cu*_x_*‐H system. Consequently, the appropriate substitution of Fe with Ni or Cu in Zr_2_Fe alloy has been demonstrated to favorably achieve the regular reaction system and significantly enhance resistance to disproportionation, without compromising the superior hydrogen storage properties. The improved modification mechanism was systematically elucidated, which may be extended to the broader development and functional optimization of Zr‐based hydrogen storage materials.

## 4 Experimental Section

### Sample Preparation

Alloy ingots with composition of Zr_2_Fe, Zr_2_Fe_1‐_ *_x_* Co*_x_* (*x* = 0.1, 0.2, 0.3, 0.4), Zr_2_Fe_1‐_ *_x_* Cu*_x_* (*x* = 0.1, 0.2, 0.3, 0.4), and Zr_2_Fe_1‐_ *_x_* Ni*_x_* (*x* = 0.1, 0.2, 0.3, 0.4, 0.5) were synthesized by induction levitation melting of high‐purity raw metals, and the substituted alloy samples were simplified as Co*x*, Cu*x*, and Ni*x* (*x* = 0.1–0.5), respectively. The equipment used was an SPG‐60AB high‐frequency induction heater produced by Shenzhen Shuangping Power Supply Technologies Co. Ltd. Stoichiometric amounts of high purity zirconium (Zr, 99.9%), iron (Fe, 99.9%), cobalt (Co, 99.9%), copper (Cu, 99.95%) and nickel (Ni, 99.9%) were melted in a water‐cooled copper crucible protected by Ar atmosphere (99.999%), and the ingots were flipped over to remelt three times to ensure high homogeneity. The prepared ingots (≈25 g) were polished to remove the oxidized surface using a grinder and then crushed into fine particles in a glove box.

### Microstructure Characterization

The morphology, elemental composition, and distribution of the samples were observed using a Hitachi SU‐8600 Field Emission Scanning Electron Microscope (FESEM) equipped with an Energy Dispersive X‐ray Spectrometer (EDS). The microstructural information was analyzed by a Tecnai G2 F20 Transmission Electron Microscope (TEM). The centered dark‐field images were obtained by first performing Selected Area Electron Diffraction (SAED) on the target region, then adjusting the objective aperture to encompass the selected diffraction spot at the center of the diffraction pattern, and finally switching back to imaging mode. The phase component and structure were characterized by an X‐pert Power X‐ray Diffractometer (XRD) with Cu‐Kα radiation (λ = 0.154056 nm). The thermal properties of hydrides were detected by a Netzsch STA449F3 Differential Scanning Calorimeter (DSC) in the Ar atmosphere at a heating rate of 10, 15, and 20 °C min^−1^. The chemical state and electronic state of alloys were acquired by an ESCALAB 250Xi X‐ray Photoelectron Spectrometer (XPS), and Ar ion sputter‐etching was used to remove surface oxides. The samples used for characterization should be ground into fine powders in a glove box and then selected by a 200‐mesh sieve to remove large particles.

### Hydrogen Storage Performance Measurements

Hydrogen storage properties tests of the samples were performed in Sievert's type volumetric equipment, which consists of a stainless‐steel reactor and two reservoirs, equipped with an electric furnace and three pressure sensors. High‐purity hydrogen (H_2_, 99.999%) was employed as the experimental gas. The crushed alloy powders were first placed in a dynamic vacuum condition at 450 °C for 1 h to get rid of the surface‐adsorbed gas impurities for activation and then used for subsequent experiments. The hydrogenation kinetics of 0.25 g activated sample was measured under initial hydrogen pressures of 0.25 bar at room temperature. In the high‐temperature hydrogenation kinetics test, with a sample loading of 0.5 g and initial hydrogen pressure and temperature adjusted to 1 bar and 450 °C, respectively. Hydrogenation pressure‐composition‐temperature (PCT) measurements of alloys were carried out at 1.0 g and detected using a sensor with an accuracy of 0.1 Pa. The high‐temperature disproportionation kinetics were conducted under an initial hydrogen pressure of 0.05 bar and at 500, 525, 550, and 575 °C, respectively.

### Theoretical Calculations

The density functional theory (DFT) calculations were conducted using the Vienna ab initio Simulation Package (VASP). The projected augmented wave (PAW) method was employed to describe the electronic exchange‐correlation interactions along with the generalized‐gradient approximation (GGA) functional in the parameterization of the Perdew‐Burke‐Ernzerhof (PBE) pseudopotential.^[^ ^50^, ^51^ ^]^ The selected models for Zr_2_Fe (mp‐1159), ZrH_2_ (mp‐24286), Zr_2_FeH_5_ (mp‐643907), Zr_3_Fe (mp‐31205), and ZrFe_2_ (mp‐1718) from the Materials Project were subjected to geometrical optimization. The plane wave cutoff energy was set to 380 eV, the self‐consistent convergence criteria of electron and ion were respectively set to be 10^−6^ eV and 0.02 eV Å^−1^, and a 5 × 5 × 6 Gamma centered Monkhorst‐Pack grid k‐points was proved to be sufficient to converge for the corresponding structure optimization. Numerical calculations of the Helmholtz free energy were performed by simple summations over phonon modes sampled on a regular grid, with M substituting for Fe at an atomic ratio of 1 to 16.^[^ ^52^ ^]^ Before calculating the substitution of Ni, a 2 × 1 × 1 cell expansion on Zr_2_FeH_5_ was performed. Then, 1, 2, and 3 Ni atoms were used as substitutes to simulate the actual experimental conditions of Zr_2_Fe_0.9_Ni_0.1_H_5_, Zr_2_Fe_0.8_Ni_0.2_H_5_, and Zr_2_Fe_0.7_Ni_0.3_H_5_.^[^ ^33^ ^]^ The most stable crystal structure of each was confirmed by calculating the lowest formation energy (Tables S5–S7, Supporting Information), and the dehydrogenation energy for each H atom was calculated based on these structures. The visualization and annotation of the models were achieved by Visualization for Electronic and Structure Analysis (VESTA).^[^ ^53^ ^]^ The chemical bonding analysis of Metal‐H interaction was carried out using the crystal orbital Hamilton population (COHP), which was calculated by the Local Orbital Basis Suite Toward Electronic‐Structure Reconstruction code (LOBSTER).^[^ ^44^, ^54^ ^]^ The integral of the crystal orbital Hamilton population (ICOHP) describes the bond strength through integrating energy below Fermi energy.^[^ ^55^, ^56^, ^57^ ^]^

## Conflict of Interest

The authors declare no conflict of interest.

## Supporting information



Supporting Information

## Data Availability

The data that support the findings of this study are available from the corresponding author upon reasonable request.
