# Supplementary material for: Achieving Anti‐Disproportionation Performance Enhancement and Distorted Inverse‐Disproportionation Reaction Correction of Zr2Fe‐Based Hydrogen Isotope Storage Alloys via Element Substitution
Source: Adv Sci (Weinh). 2025 Jun 29;12(36):e07722. doi: 10.1002/advs.202507722 (PMC12462946; doi:10.1002/advs.202507722)
Supplement: Supplementary file 1 — Supporting Information [file ADVS-12-e07722-s001.pdf]

ADVANCED SCIENCE
Open Access

Supporting Information

for *Adv. Sci.*, DOI 10.1002/advs.202507722

Achieving Anti-Disproportionation Performance Enhancement and Distorted Inverse-Disproportionation Reaction Correction of Zr_2Fe-Based Hydrogen Isotope Storage Alloys via Element Substitution

Zhiyi Yang, Yuxiao Jia, Yang Liu, Fei Chu, Jiacheng Qi, Tiao Ying, Jiahuan He, Xingwen Feng, Jiangfeng Song, Yan Shi, Wenhua Luo*, Xuezhang Xiao* and Lixin Chen**

Supporting Information

Achieving Anti-disproportionation Performance Enhancement and Distorted Inverse-disproportionation Reaction Correction of Zr_2Fe-Based Hydrogen Isotope Storage Alloys via Element Substitution

Zhiyi Yang†, Yuxiao Jia†, Yang Liu, Fei Chu, Jiacheng Qi, Tiao Ying, Jiahuan He, Xingwen Feng, Jiangfeng Song, Yan Shi, Wenhua Luo*, Xuezhang Xiao*, and Lixin Chen**

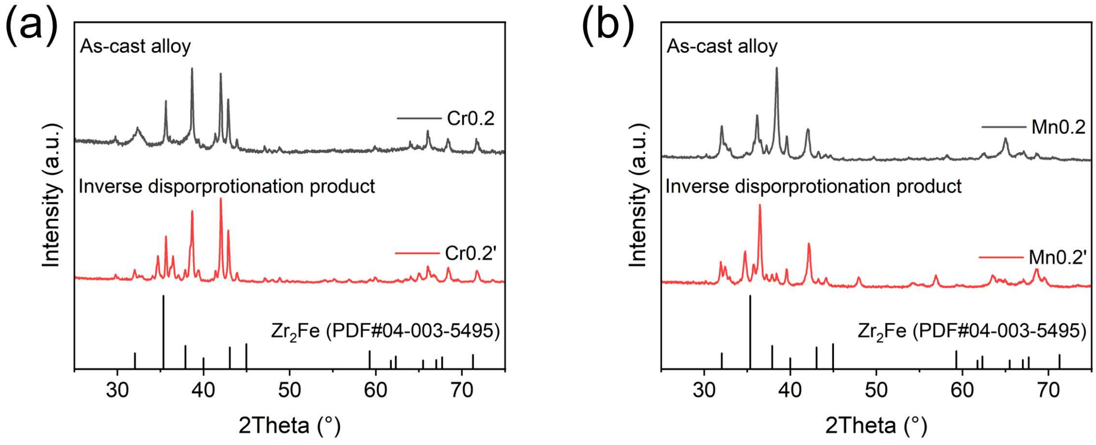

Figure S1. XRD patterns of the as-cast alloys and inverse disproportionation products: (a) $Zr_2Fe_{0.8}Cr_{0.2}$; (b) $Zr_2Fe_{0.8}Mn_{0.2}$.

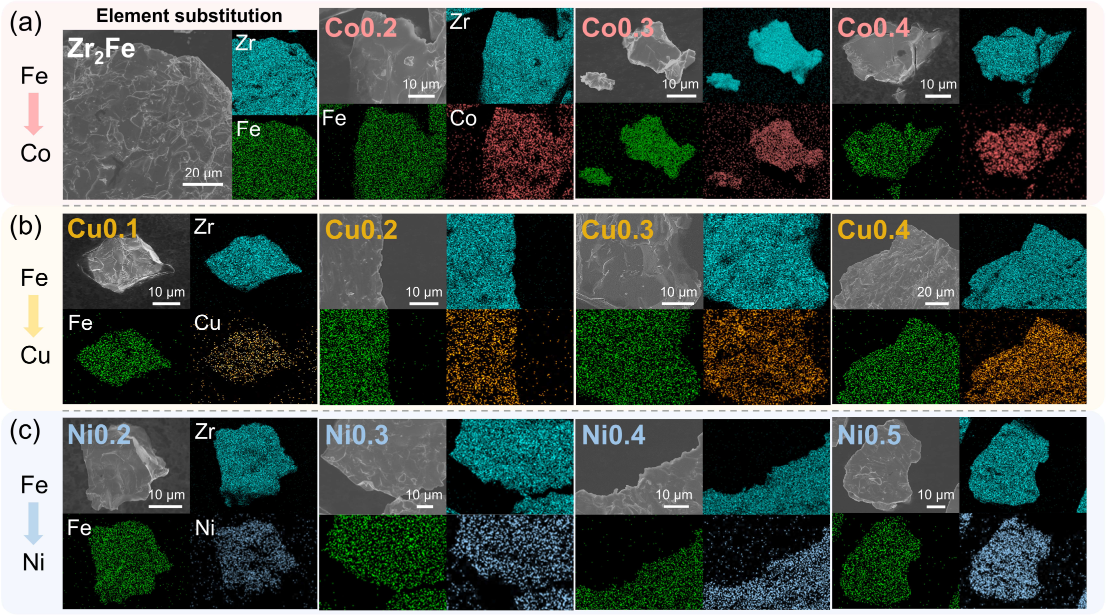

Figure S2. SEM images correlating the elemental mapping micrographs of as-cast $Zr_2Fe_{1-x}Co_x$ (a), $Zr_2Fe_{1-x}Cu_x$ (b) and $Zr_2Fe_{1-x}Ni_x$ (c) ($x = 0 \sim 0.5$) alloys.

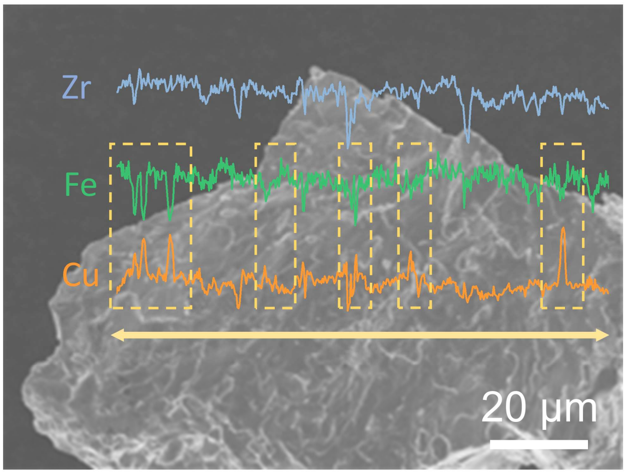

Figure S3. SEM image correlating the EDS line scanning of $Zr_2Fe_{0.8}Cu_{0.4}$ alloy.

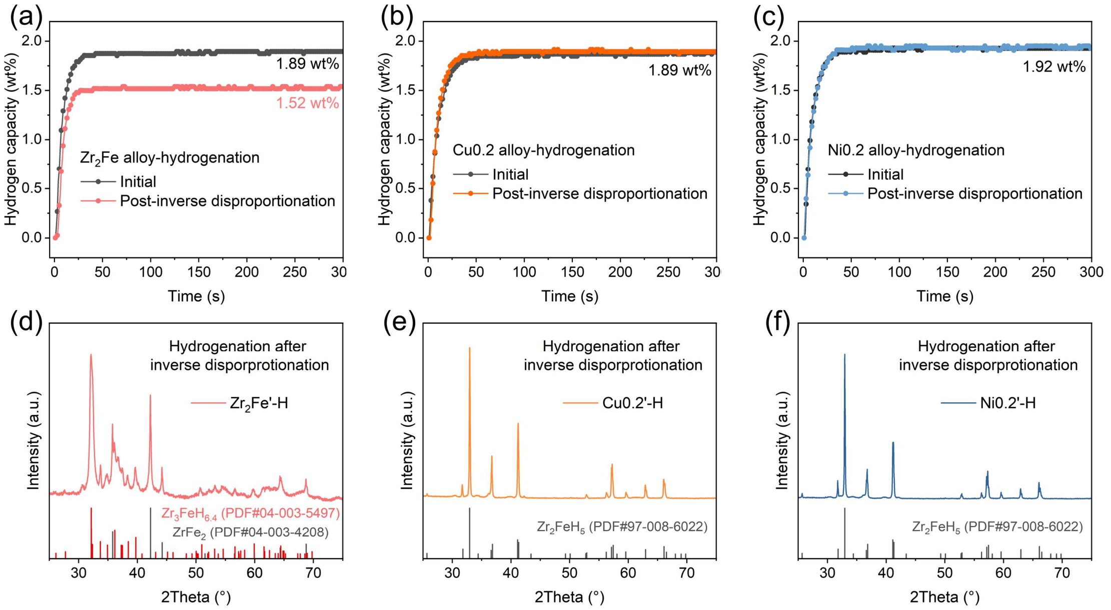

Figure S4. Initial and secondary hydrogenation kinetics under hydrogen pressure of 0.25 bar at 25 °C of Zr_2Fe, $Zr_2Fe_{0.8}Cu_{0.2}$ (a) and $Zr_2Fe_{0.8}Ni_{0.2}$ (b) alloys, and the corresponding XRD patterns of their corresponding final hydrides (d-f).

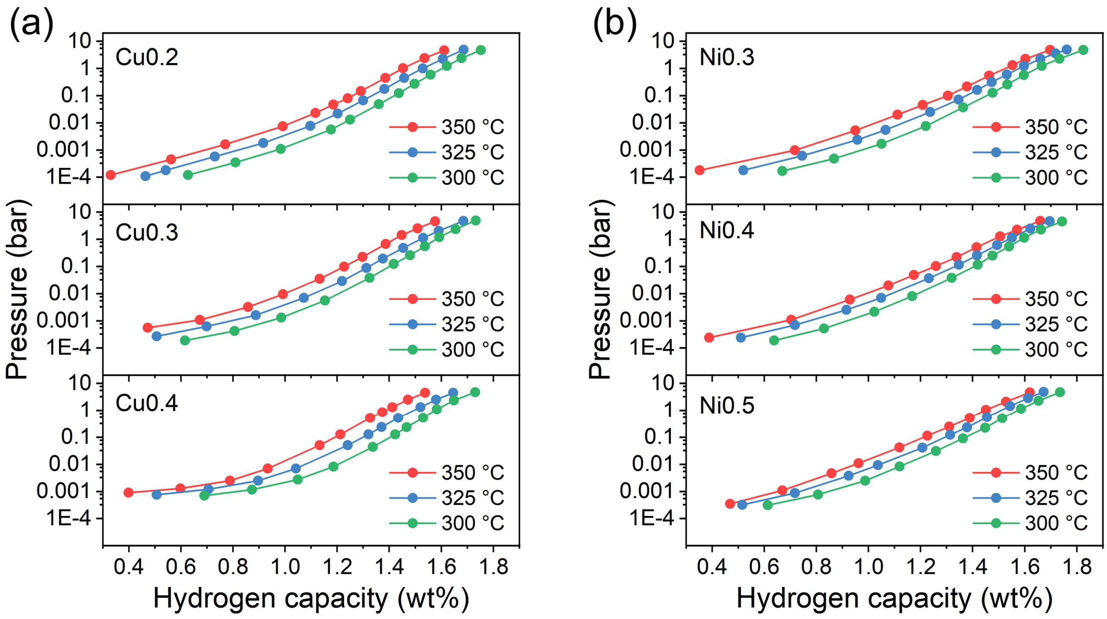

Figure S5. Hydrogenation PCT curves of $Zr_2Fe_{1-x}Cu_x$ (a) and $Zr_2Fe_{1-x}Ni_x$ (b) alloys at 300, 325 and 350 °C.

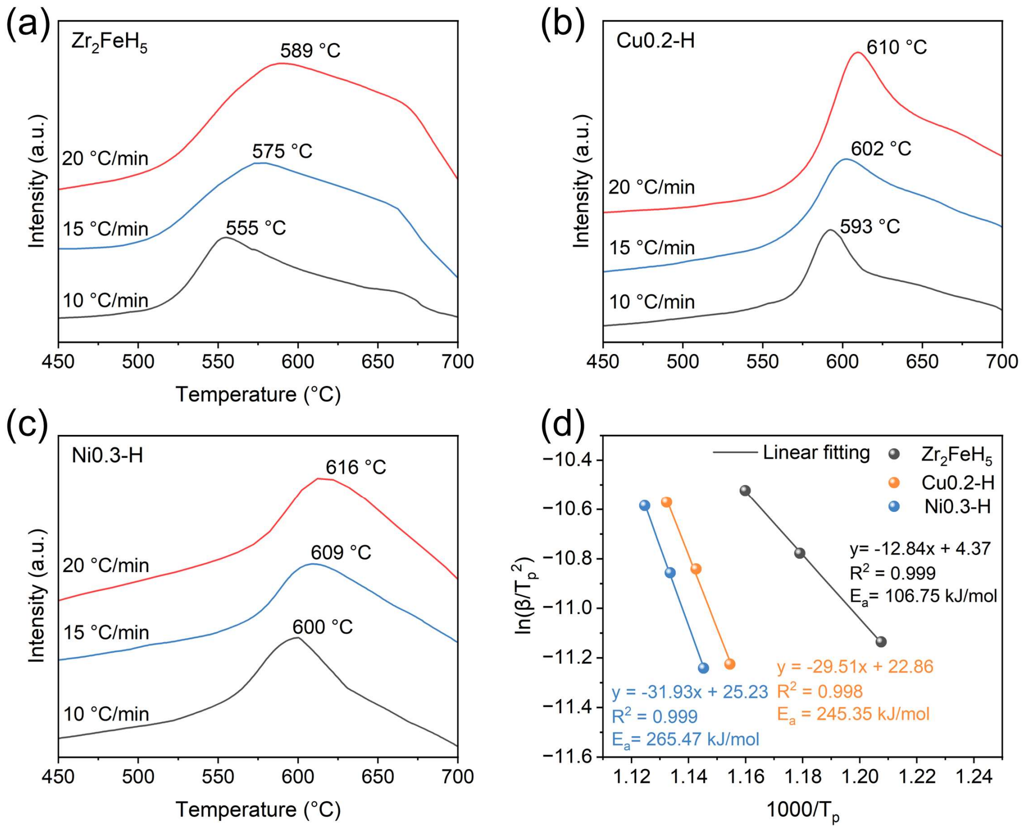

Figure S6. DSC curves of Zr_2FeH_5 (a), $Zr_2Fe_{0.8}Cu_{0.2}H_5$ (b) and $Zr_2Fe_{0.8}Ni_{0.3}H_5$ (c) hydrides at heating rate of 10, 15 and 20 °C/min, and corresponding activation energy calculation (d).

Peak temperatures of disproportionation reaction in Zr_2Fe-H, Cu0.2-H and Ni0.3-H systems were utilized to calculate the corresponding reaction activation energy (E_a) by the following Kissinger equation ($\ln(\beta/T_p^2) = -E_a/RT_p + C$), where T_p signifies the peak temperature and β denotes the heating rate; R and C are constant, R is the molar gas constant and C is related to E_a. A liner relationship can be obtained by fitting $1/T_p$ and $\ln(\beta/T_p^2)$.

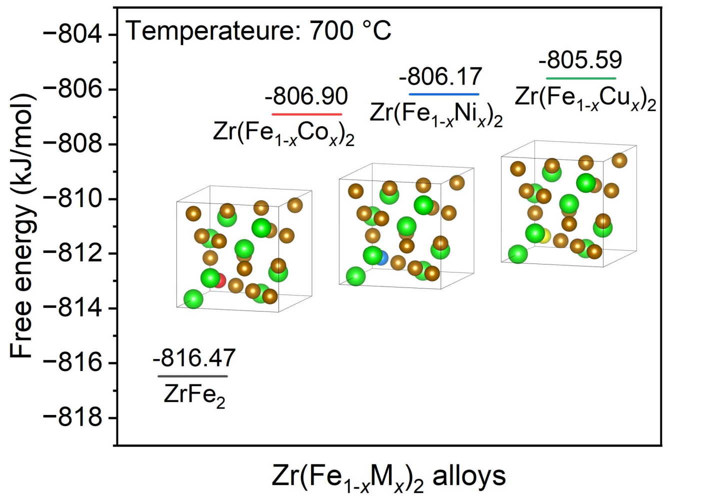

Figure S7. Free energy of $Zr(Fe_{1-x}M_x)_2$ alloys with atomic substitution of 0.0625 at 700 °C.

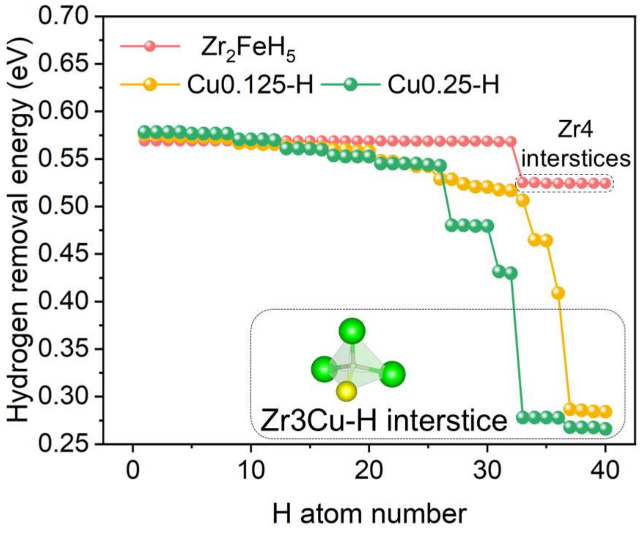

Figure S8. Hydrogen removal energies at different interstices in $Zr_2Fe_{1-x}Cu_x$-H (x = 0, 0.125, 0.25) systems.

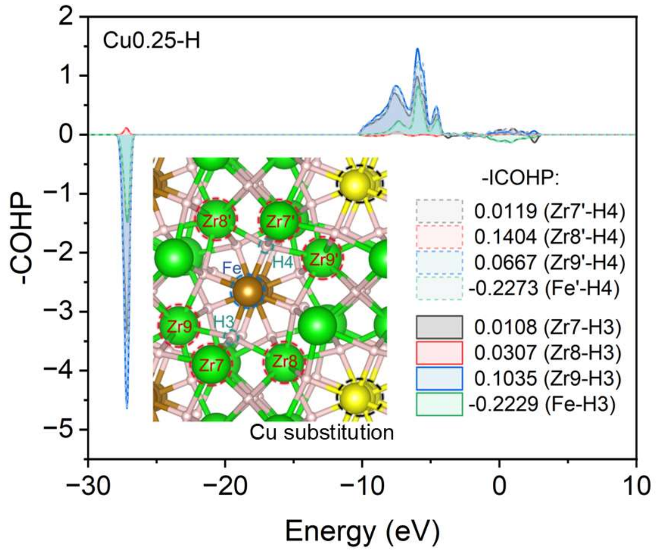

Figure S9. The Zr3Fe-H interstices and calculated COHP between Zr, Fe and H atom in Cu0.25-H.

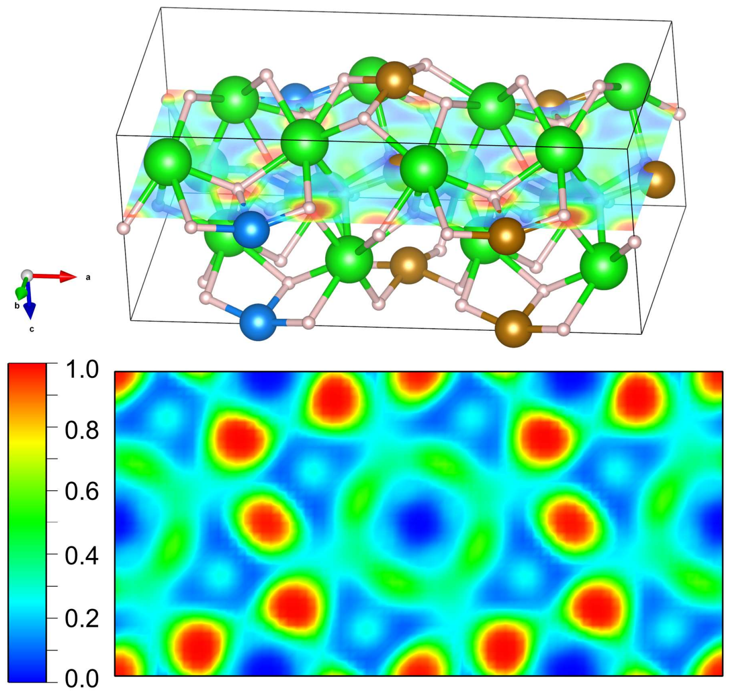

Figure S10. 2D slices diagram of electron localization function (ELF) for $Zr_2Fe_{0.75}Ni_{0.25}H_5$. The blue-yellow-red color gradient indicates the increase on intensity.

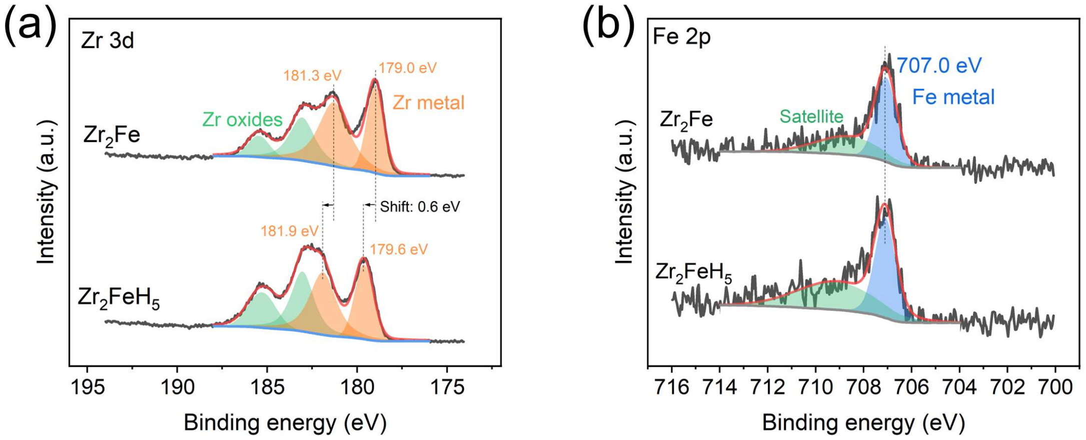

Figure S11. Zr3d (a) and Fe 2d (b) XPS spectrum of Zr_2Fe and Zr_2FeH_5 samples.

Table S1. Free energy of $Zr_2Fe_{1-x}M_x$, $Zr_2Fe_{1-x}M_x$ and $Zr(Fe_{1-x}M_x)_2$ alloys with atomic substitution of 0.0625 at 700 °C.

Free energy	Original	Co substitution	Ni substitution	Cu substitution
$F(Zr_{32}Fe_{16})$	1790.40349	1812.44974	-1817.3164	1825.54195
$F(Zr_{48}Fe_{16})$	-2504.711	2512.50177	2506.51513	2518.06992
$F(Zr_8Fe_{16})$	-816.47215	-806.90342	-806.17285	-805.58953
$\Delta F'$	-195.05985	-89.06345	-45.30909	-37.67907

The unit cell models are expanded to include the actual atomic compositions of $Zr_{32}Fe_{16}$, $Zr_{48}Fe_{16}$ and Zr_8Fe_{16}, corresponding to Zr_2Fe, Zr_3Fe and $ZrFe_2$ alloys, respectively. Accordingly, the representative reaction corresponding to $\Delta F'$ becomes equivalent to:

$$5Zr_{32}Fe_{16} == 3Zr_{48}Fe_{16} + 2Zr_8Fe_{16}$$

The corresponding free energy difference is calculated as $\Delta F' = 3\times F(Zr_{48}Fe_{16}) + 2\times F(Zr_8Fe_{16}) - 5\times F(Zr_{32}Fe_{16})$. In the substituted alloys, one Fe atom in the supercell is replaced by Co, Ni, or Cu, corresponding to an atomic substitution ratio of 1:16 (i.e., $x = 0.0625$), to form $Zr_2Fe_{1-x}M_x$, $Zr_2Fe_{1-x}M_x$ and $Zr(Fe_{1-x}M_x)_2$.

Table S2. The cross-sectional composition of Zr_2Fe, $Zr_2Fe_{1-x}Co_x$, $Zr_2Fe_{1-x}Cu_x$ and $Zr_2Fe_{1-x}Ni_x$ ($x = 0 \sim 0.5$) alloy samples.

Alloy \ Element	Zr (at%)	Fe (at%)	Co (at%)	Cu (at%)	Ni (at%)
Zr_2Fe	65.79	34.21	/	/	/
Co0.1	64.86	31.88	3.26	/	/
Co0.2	67.13	26.67	6.20	/	/
Co0.3	64.84	24.79	10.37	/	/
Co0.4	65.24	21.12	13.63	/	/
Cu0.1	65.11	31.33	/	3.56	/
Cu0.2	63.93	28.92	/	7.15	/
Cu0.3	64.84	23.70	/	11.46	/
Cu0.4	63.46	20.10	/	16.44	/
Ni0.1	66.25	30.31	/	/	3.44
Ni0.2	65.14	28.17	/	/	6.69
Ni0.3	66.02	23.59	/	/	10.39
Ni0.4	66.25	20.46	/	/	13.29
Ni0.5	65.08	17.83	/	/	17.09

Table S3. Thermodynamic characteristics of Zr_2Fe-H, $Zr_2Fe_{1-x}Cu_x$-H and $Zr_2Fe_{1-x}Ni_x$-H ($x = 0 \sim 0.5$) systems.

Alloy	P_{eq} (Pa)			ΔH (kJ/mol H_2)	ΔS (J/K·mol H_2)
	300 °C	325 °C	350 °C		
Zr_2Fe	112	305	777	-114.46	-143.23
Cu0.2	122	350	830	-113.96	-143.20
Cu0.3	155	395	1000	-110.66	-139.12
Cu0.4	215	550	1300	-106.87	-135.40
Ni0.3	117	330	800	-114.23	-143.27
Ni0.4	179	463	1100	-109.20	-137.78
Ni0.5	280	720	1600	-103.57	-131.93

Table S4. Bader charge values of specific H atoms in Zr_2FeH_5 and $Zr_2Fe_{0.875}Ni_{0.125}H_5$.

H atom in Zr3Fe-H interstices	Bader charge variation in Zr_2Fe-H (eV)	Bader charge variation in Ni0.125-H (eV)	Δ
H1	0.56916	0.57312	0.00397
H2	0.56916	0.57131	0.00215
H3	0.56916	0.57465	0.00549
H4	0.56916	0.56913	-0.00002
H5	0.56916	0.56923	0.00008
H6	0.56916	0.57466	0.00550
H7	0.56916	0.57130	0.00214
H8	0.56916	0.57302	0.00386
H9	0.56916	0.56902	-0.00013
H10	0.56916	0.56998	0.00083
H11	0.56916	0.57260	0.00345
H12	0.56916	0.57259	0.00344
H13	0.56916	0.56999	0.00083
H14	0.56916	0.56901	-0.00014
H15	0.57157	0.57010	-0.00147
H16	0.57157	0.57007	-0.00150
H17	0.57157	0.56784	-0.00374
H18	0.57157	0.57458	0.00300
H19	0.57157	0.57456	0.00299
H20	0.57157	0.56785	-0.00373
H21	0.57157	0.57006	-0.00152
H22	0.57157	0.57011	-0.00146
H23	0.57157	0.56996	-0.00161
H24	0.57157	0.57107	-0.00050
H25	0.57157	0.57060	-0.00098
H26	0.57157	0.57003	-0.00154
H27	0.57157	0.57108	-0.00050
H28	0.57157	0.56995	-0.00162
Average (H1-H28)	0.57036	0.57098	

Table S5. Static energy values of $Zr_2Fe_{0.875}Ni_{0.125}H_5$ with Ni substitution at different site.

Substitution site	*E*(eV)
Ni1	-361.75445111
Ni2	-361.75378546
Ni3	-361.75365269
Ni4	-361.75413724
**Ni5**	**-361.75460260**
Ni6	-361.75412024
Ni7	-361.75452562
Ni8	-361.75452333

Table S6. Static energy values of $Zr_2Fe_{0.75}Ni_{0.25}H_5$ with Ni substitution at different site.

Substitution site	*E*(eV)	Substitution site	*E*(eV)
Ni1	-359.1134460400	Ni15	-359.1408196600
**Ni2**	**-359.2716570800**	Ni16	-359.1405470700
Ni3	-359.1396368200	Ni17	-359.1595672000
Ni4	-359.1599442700	Ni18	-359.1599021200
Ni5	-359.1604103800	Ni19	-359.1407805500
Ni6	-359.1415751700	Ni20	-359.1429358200
Ni7	-359.1421848100	Ni21	-359.1614370600
Ni8	-359.1356747900	Ni22	-359.1604326700
Ni9	-359.2712224400	Ni23	-359.1146841500
Ni10	-359.1620764900	Ni24	-359.2714004000
Ni11	-359.1605987000	Ni25	-359.1374026300
Ni12	-359.1424715000	Ni26	-359.1373611500
Ni13	-359.1408976400	Ni27	-359.2711766300
Ni14	-359.1120764200	Ni28	-359.1149375700

Table S7. Static energy values of $Zr_2Fe_{0.625}Ni_{0.375}H_5$ with Ni substitution at different site.

Substitution site	*E*(eV)	Substitution site	*E*(eV)
Ni1	-356.6639020600	Ni29	-356.7240766200
Ni2	-356.6637505000	Ni30	-356.7238010200
Ni3	-356.5730852800	Ni31	-356.5726533900
Ni4	-356.5726079000	Ni32	-356.7238777900
Ni5	-356.5455343700	Ni33	-356.5797546400
Ni6	-356.5435702700	Ni34	-356.5797636900
Ni7	-356.6639618200	Ni35	-356.7232609500
Ni8	-356.7237944500	Ni36	-356.5434002500
**Ni9**	**-356.7253181200**	Ni37	-356.5448171300
Ni10	-356.7236643600	Ni38	-356.5457574100
Ni11	-356.7240490700	Ni39	-356.5712755900
Ni12	-356.5791678200	Ni40	-356.5726679100
Ni13	-356.5805199900	Ni41	-356.5454643900
Ni14	-356.5801958400	Ni42	-356.7236913600
Ni15	-356.5792080600	Ni43	-356.5789834500
Ni16	-356.5725307700	Ni44	-356.5797632100
Ni17	-356.7231209400	Ni45	-356.7233108100
Ni18	-356.5797624900	Ni46	-356.5724681700
Ni19	-356.5797530600	Ni47	-356.5453160200
Ni20	-356.7237705900	Ni48	-356.7242907700
Ni21	-356.5458767800	Ni49	-356.5796848900
Ni22	-356.6660071700	Ni50	-356.5797568600
Ni23	-356.5790021000	Ni51	-356.7231691800
Ni24	-356.5790008200	Ni52	-356.5719007800
Ni25	-356.5792446400	Ni53	-356.6649086300
Ni26	-356.5789911200	Ni54	-356.6648825900
Ni27	-356.7236549400	Ni55	-356.6664150400
Ni28	-356.7236745600	Ni56	-356.6661030400
